# A hybrid encryption framework leveraging quantum and classical cryptography for secure transmission of medical images in IoT-based telemedicine networks

**DOI:** 10.1038/s41598-024-82256-3

**Published:** 2024-12-28

**Authors:** Arslan Shafique, Syed Ali Atif Naqvi, Ali Raza, Masoud Ghalaii, Panagiotis Papanastasiou, Julie McCann, Qammer H. Abbasi, Muhammad Ali Imran

**Affiliations:** 1 School of Electronic and Nanoscale Engineering, University of Glasgow, Glasgow, G12 8QQ UK; 2 School of Electronic and Electrical Engineering, University of Leeds, Leeds, LS2 9JT UK; 3 Department of Computer Science, University of York, York, YO10 5DD UK; 4 Department of Computing, Imperial College, London, SW7 2AZ UK

**Keywords:** Image processing, Computer science, Information technology

## Abstract

In the era of the Internet of Things (IoT), the transmission of medical reports in the form of scan images for collaborative diagnosis is vital for any telemedicine network. In this context, ensuring secure transmission and communication is necessary to protect medical data to maintain privacy. To address such privacy concerns and secure medical images against cyberattacks, this research presents a robust hybrid encryption framework that integrates quantum, and classical cryptographic methods. The proposed framework not only secure medical data against cyber threats but also protects the secret security keys. Initially, a Quantum Key Distribution (QKD) is employed to generate a shared key, which is then used to secure the symmetric keys via One-Time Pad (OTP) encryption. Next, bit-planes are extracted from each color component. The rows and columns of the extracted bit-planes are scrambled using random sequences which are generated by a 6D hyperchaotic Chen system and the Ikeda map. To further increase confusion in the original data, multiple-step pixel scrambling operations such as pixel shuffling, pixel value shuffling, and rotational and flipping operations are implemented. After the confusion phase, a combination of affine transformations with non-linear functions, Discrete Cosine Transform (DCT) with complex modulation, Discrete Wavelet Transform (DWT) with random phase modulation, bilinear transformation, and nonlinear polynomial mapping are employed to create diffusion in the scrambled components. These multiple encryption operations aim to maximize randomness in the final ciphertext image. Additionally, to reduce computational complexity, only the Most Significant Bit-Planes (MSBs) are encrypted, as they contain more than 94% of the plaintext information. Several experimental results and analyses are conducted to assess the proposed encryption framework, including entropy analysis, key sensitivity analysis, correlation analysis lossless analysis, and histogram analysis. Furthermore, the framework is tested against various cyberattacks such as brute-force attacks, clipping attacks, and noise attacks on the ciphertext images, to demonstrate its resilience against such threats.

## Introduction

The evolution of the Internet from digitization to intellectualization is transforming daily life and industry^1^. As the transfer of digital data, specifically digital images, occurs in huge amounts between Internet of Things (IoT) devices, it is necessary to secure them from eavesdropper access. Developing a robust technology to secure digital images is not enough because IoT devices such as smartphones and tablets are very resource-constrained, including limited power and limited storage^2^. As digital images contain a huge amount of data with a high correlation between image pixels, while developing algorithms for securing digital data, it should be kept in mind that they should be capitalised with such IoT devices having limited resources.

To protect image information from unauthorised access, three main technologies, such as encryption methods and steganographic techniques, and techniques for securing encryption secret keys have been frequently used in the past several years^3–5^. Image encryption includes several approaches such as frequency domain encryption^6^, spatial domain encryption, compressed sensing^7^, and encryption based on optical and quantum computing^8,9^. Spatial domain encryption modifies pixel values directly, while transform-based encryption uses transformations to alter pixel values in both the spatial and transform domains. Compressive sensing incorporates both compression and encryption to enhance the processing time while also increasing the chances of losing data due to decryption. A new image encryption approach that enhances the use of encryption icing with optical properties^10^. Quantum image encryption, on the other hand, employs quantum encoders to secure digital images from cyber threats^11^. It is worth noting that encryption methods based on quantum phenomena are rather superior to those classical methods, which are logistic in nature. Most of the encryption techniques have a positive impact depending on the purpose of use^12–15^. For example, the advanced encryption standard (AES)^16^ is very effective when enhanced privacy is needed over digital pictures, but when it comes to IoT devices, it is not suitable as it has multiple encryption rounds and also includes very high-resolution encrypted images that take large storage space. However, with the growth in quantum computing, even the most secure algorithms like AES could become vulnerable.

To overcome the vulnerabilities related to the high latency and weak security, quantum operations such as quantum scrambling, and quantum substitution can be utilized. The scrambling process rearranges positions of the image pixel using random numbers generated by methods such as Blum Blum Shub^17^, chaotic systems^7,11,18^, ChaCha20^19^, and Random Number Generation Uniform (RANDU)^20^. Among them, chaotic systems are very reliable due to their robust properties, such as sensitivity to initial conditions, aperiodicity, topological mixing, and ergodicity. However, pixel permutation alone does not provide strong security for images, as it only rearranges pixel positions without altering the pixel values themselves^21^. Combining scrambling with diffusion provides stronger encryption by reducing the image pixel correlations. An effective encryption should use both permutation and diffusion for strong protection. Traditional encryption methods such as AES, and DES are unsuitable for real-time applications like video conferencing due to limited efficiency and security. However, quantum image encryption provides enhanced security and efficiency by leveraging quantum mechanics.

In this research, a robust and time-efficient hybrid quantum encryption framework is proposed. The proposed encryption framework utilizes QKD for key management, where a shared key is generated through QKD and then encrypted using a One-time Pad (OTP). This combination ensures that the secret keys are highly secure which makes it virtually impossible for eavesdroppers to recover the original secret keys. Moreover, the encryption process begins by extracting the R, G, and B components from the plaintext image. Bit-planes are then extracted from each color component of the input image. As most of the plaintext information present in the Most Significant Bit Planes (MSBPs)^22^, therefore, only these bit-planes are selected for encryption to reduce the computational complexity of the proposed encryption framework. The remaining, unencrypted bit-planes are simply combined to form pre-ciphered images for each color component. For the encryption of the MSBPs, random sequences are generated using specific initial conditions and control parameters of the chaotic maps such as 6D hyper Chen chaotic map(6DHCCM), and Ikeda map that act as secret keys. These sequences are employed to perform three types of scrambling: pixel scrambling, pixel value permutation, and rotation and flipping. Additionally, the process of generating random sequences is extended to enhance the diffusion process using XOR operation. Further diffusion is achieved by applying a variety of operations, including Affine transformations with nonlinear functions, discrete cosine transform (DCT), wavelet transform with random phase modulation, bilinear transformation with complex weights, and nonlinear polynomial mapping to produce the final encrypted image. The contributions of the paper are as follows:

- A novel hybrid encryption framework is proposed that combines quantum and classical cryptographic techniques developed to secure the transmission of medical images in IoT-based telemedicine networks.
- In the proposed work, QKD is combined with OTP encryption to generate shared keys and secure the symmetric secret keys, which enhances the key management process.
- To reduce the computational complexity, bit-planes are extracted from the color components of the medical images and scramble only such bit-planes that contain the majority of the image information (over 94%) using random sequences generated by a 6D hyperchaotic Chen system and the Ikeda map.
- Instead of using a single scrambling step, three-step pixel scrambling operations such as pixel shuffling, pixel value shuffling, and rotational and flipping operations are applied to create randomness in the plaintext image.
- In the diffusion phase, affine transformations with non-linear functions, DCT with complex modulation, DWT with random phase modulation, bilinear transformations, and nonlinear polynomial mapping are combined to maximize randomness in the encrypted images.

The remainder of the paper is structured as follows: Section 2 offers a brief summary of existing works including their advantages and vulnerabilities. Also, this section provides the advantages, vulnerabilities, and possible solutions to overcome such vulnerabilities. Section 3 delivers a comprehensive explanation of the foundational knowledge necessary for the development of the proposed encryption framework. Section 4 presents a step-by-step explanation of the proposed encryption framework. In Section 5, the experimental results and analysis of the proposed encryption framework are discussed. Section 6 presents a concise discussion of the entire proposed work, along with an overview of the advantages of utilizing multiple encryption layers within the framework. Finally, Section 7 concludes the entire proposed research, highlights the limitations of the proposed work, and also provides a few recommendations for future research to address these limitations.

## Related work

The development of image encryption techniques has seen substantial progress, incorporating contemporary technologies to enhance data security. This section explores the existing encryption schemes and investigates their vulnerabilities. For instance, In^23^, Hu et al. proposed a quantum image encryption method using a qubit-level scrambling^23,24^. This approach enhances operational effectiveness by the use of hyperchaotic states and sometimes controlling periodic windows, while the diffusion and scrambling methods aid image encryption further. Quite, the scheme is not effective if the parameters of the chaotic maps and quantum model are not efficiently selected.

In^25^, He et al. proposed a quantum image encryption algorithm, that enhances quantum image preparation by optimizing quantum circuits to reduce auxiliary qubits. The algorithm also deals with non-random key issues with a modified logistic map^26^and enhances security with row-column-based permutation^27^. However, vulnerabilities may arise if the circuit optimization or permutation methods are exploited, and the encryption could be weakened if the improved logistic map lacks adequate randomness or security. In^28^, Mohamed et al. proposed a quantum-based framework for the encryption of digital images. They perform pixel diffusion and pixel permutation by using quantum operations like Hadamard gates^29^and quantum-controlled gates^30^and perform key generation using a hyperchaotic system, bloomschaotic sequences, and circular shits for permutation. This scheme poses a risk of attacks owing to the hyperchaotic being predictable. In^31^, Gao et al. proposed quantum DNA-based cryptographic techniques. To round off the pixel transformation images of the new frame, alternation of colours of particular pixels within the images is achieved using Hilbert scrambling^30^. Their scheme is vulnerable to attacks due to weak management of the inverse operations and the key matrix. In^32^, Zhang et al. presented quantum image cryptosystems that include superposition states in reversible pixel scramblers. It also incorporates a pixel reordering feature in which a feature-orientated order of the image is processed with sequential cries. In^33^, Wen et al presented an encryption scheme based on chaos-based block scrambling and confusion-diffusion operations. It encrypts the digital images using multiple cryptographic operations, such as block-wise scrambling, and rotation. It also satisfies cryptographic criteria for confusion, diffusion, and avalanche effects.

In^34^, Rehman et al. proposed an encryption framework for the protection of color image schemes dependent on OTP keys and chaos theory with a rotor machine concept. The image pixel rows and columns are transformed and rotated to create new configurations for substitution. Vulnerabilities arise in their scheme due to the predictable patterns of the rotor rotation and chaotic sequences. Moreover, the one-time keys and chaotic maps are not securely managed or sufficiently randomized. In^35^, Kumar et al. introduced an encryption scheme that converts digital images into a 2D difference matrix using a median edge detector. The image is then encoded with a bit-plane representation to reduce the size and enable the embedding more secret data. In^36^, Zhu et al. performed cryptanalysis of encryption frameworks using bit plane extraction to find the key streams for bit-level permutation and XOR diffusion. This independence allows attackers to recover the permutation and diffusion keys through chosen plaintext attacks using just two specific plaintext images and their ciphertexts, exposing a significant vulnerability in the encryption system. In^37^, Sing et al. presented a chaotic system based encryption scheme in which key is initialized with confusion- diffusion operations. While the method offers strong security and high speed, it is not enough to resist cyberattacks because of the exploitable patterns in the object detection process. A summary of the existing works is provided in Table 1.

**Table 1:** Summary of Image Encryption Schemes.

**Existing schemes**	**Application domain**	**Real-World performance**	**Robustness against attacks**	**disadvantages**	**Potential solutions**
Hu et al.^23^	Quantum Image Encryption	Enhanced security	Vulnerable to poor parameters	Chaotic maps vulnerabilities	Improve parameter selection
He et al.^25^	OCPBP Quantum Encryption	Optimized BRQI prep	Circuit and key randomness issues	Weak circuit optimization	Better optimization techniques
Mohamed et al.^28^	Quantum Cellular Automata	Effective diffusion	Predictable hyperchaotic system	Predictable chaos	Use less predictable chaos
Gao et al.^31^	Quantum DNA Code	Uses Hilbert scrambling	Weak key management	Weak key matrix	Improve key management
Zhang et al.^32^	Quantum State Superposition	Large key space	Vulnerable to attacks	Weak state transmission	Enhance state security
Wen et al.^33^	Chaos-Based Block Permutation	Satisfies criteria	Not detailed	Not addressed	Further robustness evaluation
Rehman et al.^34^	Color Image Encryption	Transforms pixel rows	Predictable rotor patterns	Weak key management	Secure key management
Kumar et al.^35^	Bit-Plane Representation	Effective embedding	Not detailed	Limited robustness details	Enhance robustness evaluation
Zhu et al.^36^	Bit Plane Extraction	Key streams analyzed	Vulnerable to attacks	Exploitable key recovery	Improve key management
Singh et al.^37^	Chaos-Based for Smart Cities	Fast object detection	Exploitable detection patterns	Patterns in detection	Better detection security

## Preliminaries

In the proposed research, several key components are used, and it is essential to understand their importance and roles within the framework. The following subsections provides a brief overview of these critical encryption elements and explain why they are integral to the proposed encryption approach.

### 6D Hyperchaotic Chen system

**Fig. 1:**
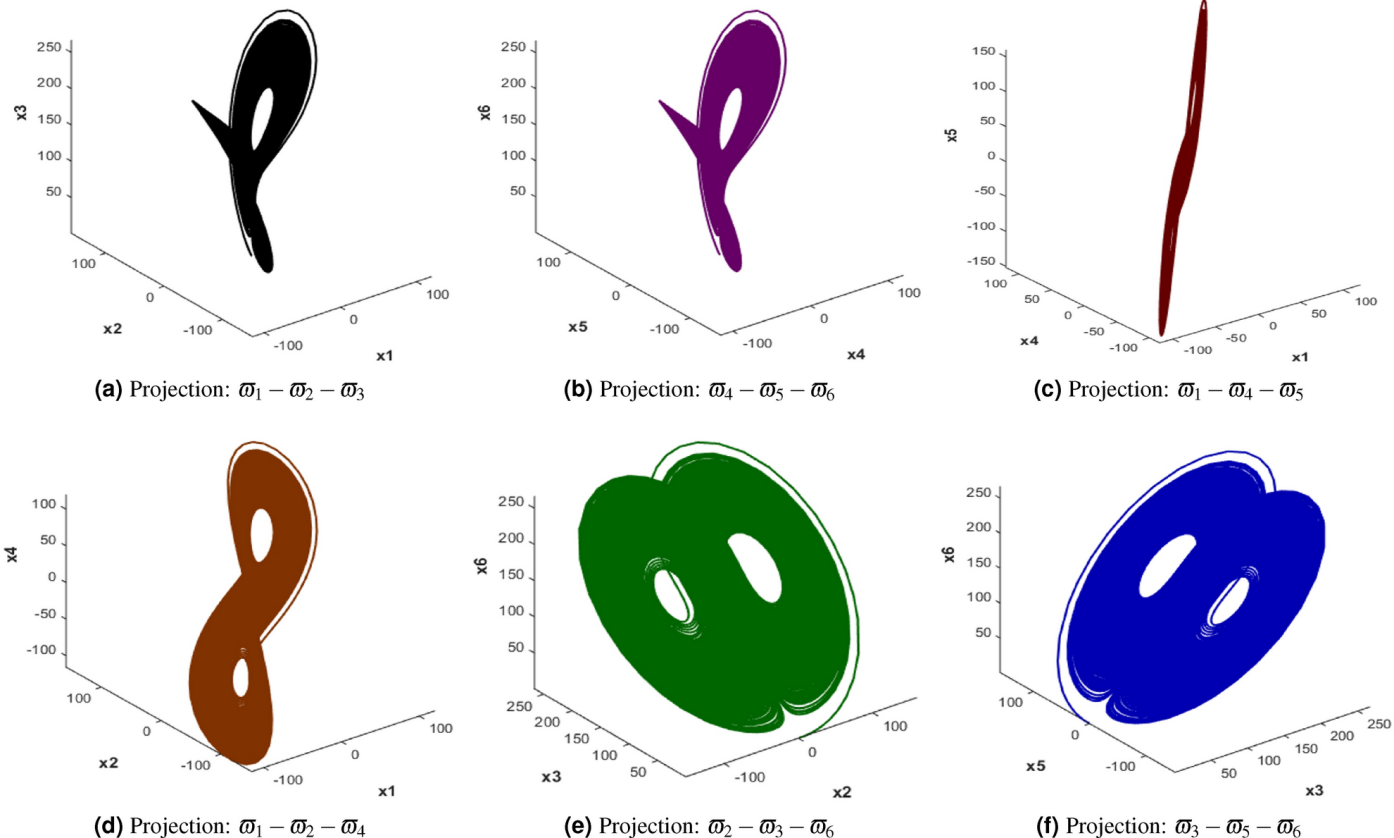
6D Hyperchaotic Chen system attractors.

The 6D Hyperchaotic Chen system (6DHCCS)^38^ is a higher-dimensional extension of the classic Chen system which is designed to exhibit complex and extremely chaotic behavior. The additional dimensions in this system increase its complexity and unpredictability which makes it suitable for secure communications and cryptography applications. Mathematically, 6DHCCS can be expressed using Equation 1.

1 $$\begin{aligned} \begin{aligned} \dot{\varpi }_1&= \sigma _1 (\varpi _2 - \varpi _1) \\ \dot{\varpi }_2&= \rho _1 \varpi _1 - \varpi _2 - \varpi _1 \varpi _3 \\ \dot{\varpi }_3&= -\beta _1 \varpi _3 + \varpi _1 \varpi _2 \\ \dot{\varpi }_4&= \sigma _2 (\varpi _5 - \varpi _4) \\ \dot{\varpi }_5&= \rho _2 \varpi _4 - \varpi _5 - \varpi _4 \varpi _6 \\ \dot{\varpi }_6&= -\beta _2 \varpi _6 + \varpi _4 \varpi _5 \end{aligned} \end{aligned}$$

where $\varpi _1, \varpi _2, \varpi _3, \varpi _4, \varpi _5, \varpi _6$ are the state variables, and $\sigma _1, \rho _1, \beta _1, \sigma _2, \rho _2, \beta _2$ are the system parameters. In 6D, visualization can be challenging, but projections of the attractor into lower-dimensional spaces (such as 3D) reveal intricate and complex trajectories as shown in Figure 1. By analyzing various 3D projections of the system-such as $\varpi _1-\varpi _2-\varpi _3, \varpi _4-\varpi _5-\varpi _6$, and combinations involving other dimensions, it can be seen that each projection reveals unique patterns and interactions among the state variables, which can be utilized to generate diverse and unpredictable pseudorandom sequences for encrypting image pixels. Moreover, 3D projections of the 6D hyperchaotic Chen system highlight its complex trajectories and high sensitivity, which enable it to disrupt predictable image information when used in pixel scrambling.

The values of the state variables $\varpi _1, \varpi _2, \varpi _3, \varpi _4, \varpi _5, \varpi _6$ are obtained through numerical integration of the 6D Hyperchaotic Chen System’s differential equations, and these values are computed over time and are not fixed but rather evolve according to the system dynamics. However, the values of $\sigma _1, \rho _1, \beta _1, \sigma _2, \rho _2, \beta _2$ are 70, 150, 20, 70, 150, and 20, respectively.

### Ikeda map

**Fig. 2:**
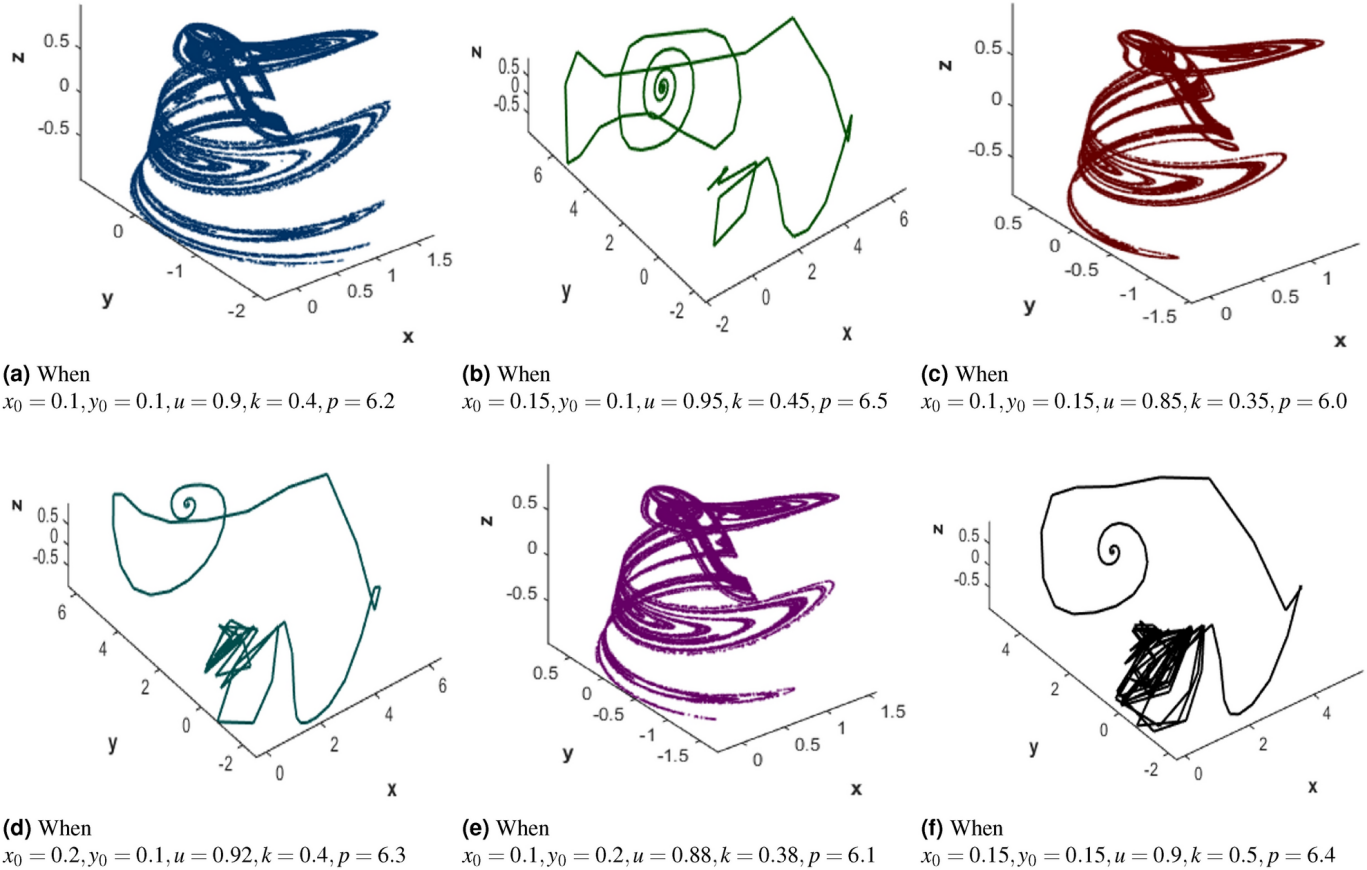
3D Ikeda map attractors demonstrating chaos and sensitivity.

The Ikeda map is a discrete dynamical system that models the behavior of light in an optical cavity with a nonlinear medium^39^. Equation 2 is the mathematical representation of Ikeda map.

2 $$\begin{aligned} \begin{aligned} \Xi _{n+1}&= 1 + u (x_n \cos (\theta _n) - \xi _n \sin (\theta _n))\\ \xi _{n+1}&= u (\Xi _n \sin (\theta _n) + \xi _n \cos (\theta _n)) \end{aligned} \end{aligned}$$

where: $\Xi _n$ and $\xi _n$ are the coordinates of the point at the $n^{th}$ iteration. $u$ controls the behavior of the map, and $\theta _n$ represents the phase shift at the $n$-th iteration as given in Equation 3.

3 $$\begin{aligned} \theta _n = k - \frac{p}{1 + x_n^2 + y_n^2} \end{aligned}$$

where $k$ and $p$ control the nonlinearity and feedback in the system. Figure 2(a-f) illustrates the six attractors of Ikeda map attractors under different initial conditions that demonstrate its chaotic behavior and sensitivity, which produce non-repeating sequences for further scrambling and diffusion of image data. Together, these chaotic systems can be used to create strong randomness in the encrypted image to enhance security by concealing any identifiable structure and making it difficult for attackers to retrieve the original image.

## Proposed encryption process

**Figure 3:**
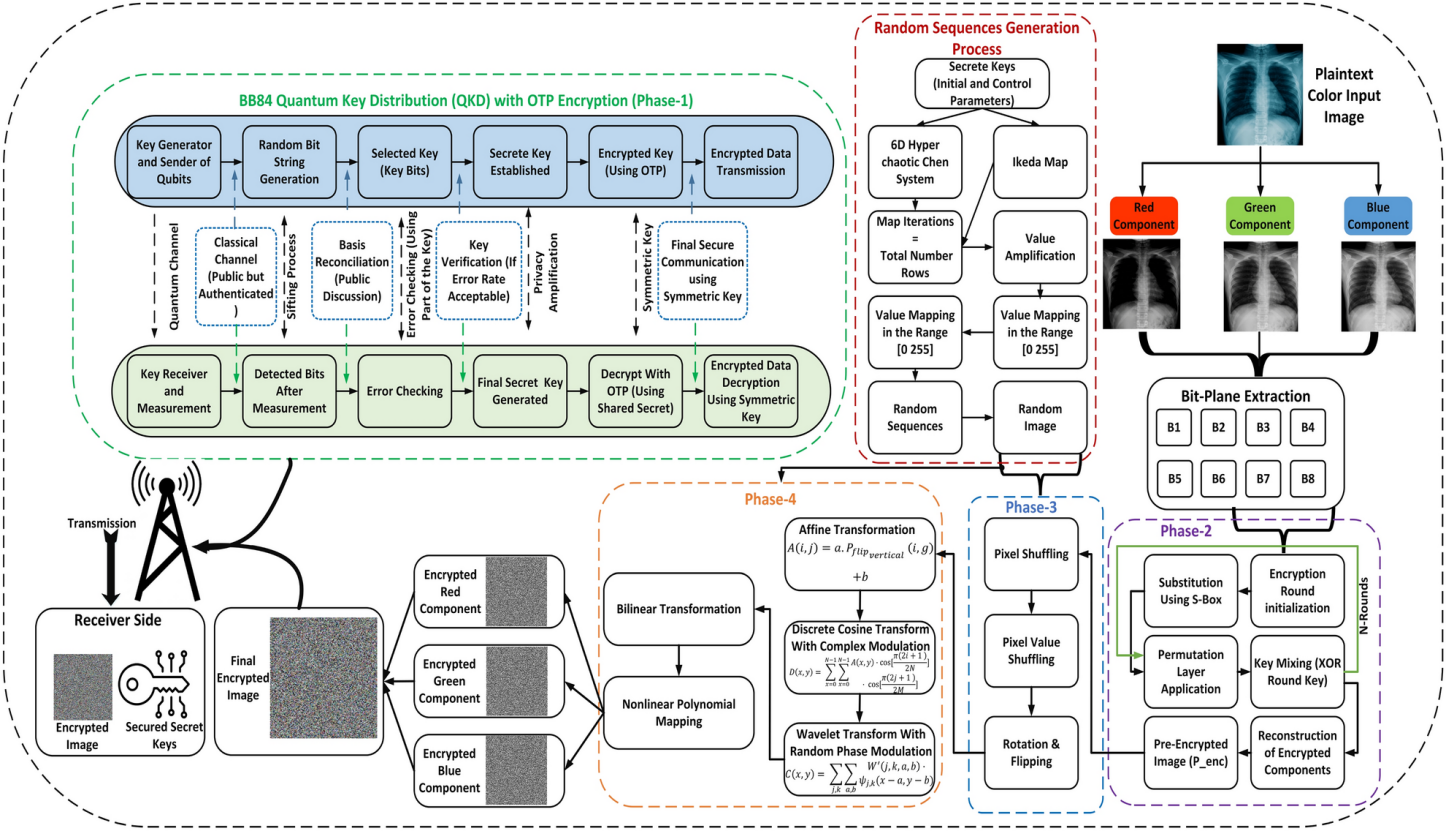
Propped encryption framework.

The proposed encryption process includes four main phases: (i) Generating a shared key using QKD with OTP encryption, (ii) extracting bit-planes from each color component and encrypting them through scrambling operations using random sequences generated by the 6DHCCS and the Ikeda map, (iii) applying confusion operations through multiple permutation techniques, and (iv) achieving diffusion in the scrambled image. A detailed overview of the entire encryption framework is illustrated in Figure 3. Each step is explained in detail in the following subsections.

The proposed encryption process consists of four key phases: (i) Generating of the shared key using QKD which is further based on OTP encryption chaotic systems, (ii) bit-planes extraction from each color component, and encrypting using scrambling operations applied based on the random sequences generated using 6DHCCS, and Ikeda map, (iii) carrying out confusion operations using multiple permutation operations, and (iv) creating diffusion in the scrambled image. The details of each step are provided in the next subsections.

### QKD based symmetric key sharing

Our proposed encryption framework begins with the implementation of QKD for generating secret keys. QKD allows multiple parties to generate shared secret cryptographic keys, which can be used for secure communication. In the proposed research, the BB84 algorithm, which is proposed by Charles Bennett and Gilles Brassard^40^, is used to secure the actual symmetric keys that are used in the generation of the chaotic sequences.

Alice randomly prepares a sequence of quantum bits (qubits), where each qubit can be in one of four possible states, based on two different bases: Rectilinear ($+$) and Diagonal ($\times$). The rectilinear ($+$) and the diagonal ($\times$) basis states are $|1\rangle \quad \text {(bit value 1)}$, $|1\rangle \quad \text {(bit value 1)}$ and $|+\rangle = \frac{1}{\sqrt{2}}(|0\rangle + |1\rangle ) \quad \text {(bit value 0)}, |-\rangle = \frac{1}{\sqrt{2}}(|0\rangle - |1\rangle ) \quad \text {(bit value 1)}$, respectively.

Now Alice prepares a sequence of 8 qubits. For this example, bit sequence (b) is $(1, 0, 1, 1, 0, 1, 0, 0)$, and basis choice ($b_B$) are $(+, \times , +, \times , +, \times , +, \times )$. Here, the Rectilinear basis $+$ is represented as $|0\rangle$ and $|1\rangle$, and the Diagonal basis $\times$ is represented as $|+\rangle$ and $|-\rangle$.

Bob randomly chooses his measurement basis. For instance, Bob’s basis ($$b_{B'})$$ are $$(\times$$, +, +, $$\times$$, $$\times$$ +, $$\times$$, + ). Now Bob will measure the qubits and obtain the following results as mentioned in Table 2

**Table 2:** Bob’s basis choice and measurement.

Bits	Qubits	Measured results
1.	Qubit 1	Measured in $\times$ basis, Bob gets $1$ (correct basis)
2.	Qubit 2	Measured in $+$ basis, Bob gets$0$ (correct basis)
3.	Qubit 3	Measured in $\times$ basis, Bob gets nothing (incorrect basis)
4.	Qubit 4	Measured in $+$ basis, Bob gets$0$ (correct basis)
5.	Qubit 5	Measured in $\times$ basis, Bob gets $0$ (correct basis)
6.	Qubit 6	Measured in $+$ basis, Bob gets $1$ (correct basis)
7.	Qubit 7	Measured in $\times$ basis, Bob gets $1$ (correct basis)
8.	Qubit 8	Measured in $\times$ basis, Bob gets nothing (incorrect basis)

Now Alice and Bob publicly compare their chosen bases and retain only those bits where their bases match.

- **Alice’s Bases:** $$(+, \times , +, \times , +, \times , +, \times )$$
- **Bob’s Bases:** $$(\times , +, \times , +, \times , +, \times , +)$$

The retained bits will be 1,0,0,0,1,1. Therefore, Alice and Bob both have the shared key of 1,0,0,0,1,1. No, the following procedure is used to encrypt the symmetric key.

- **Define the symmetric key:** Assume $\alpha = 3.49$. Convert $\alpha$ to binary, which is $\alpha _{\text {binary}} = 11.011111$.

Suppose a scaling factor of 100 is used (two decimal places), $3.49 \times 100 = 349$ $\rightarrow$ - $349$ in binary is $101011101_2$. Now pad the binary of 100011 to match the length of 101011101:

$$\begin{aligned} \begin{array}{ccccccccc} 1& 0& 1& 0& 1& 1& 1& 0& 1 \\ 0& 0& 0& 1 & 0 & 0 & 0 & 1 & 1 \\ \hline 1& 0& 1& 1& 1& 1& 1& 1& 0 \\ \end{array} \end{aligned}$$

This merged output (M) is sent over the public channel. The receiver, knowing both the shared key and the merged output, performs XOR again to recover the original symmetric key $\alpha$. This ensures that even if the merged output is intercepted by the eavesdropper, without the shared key, it is impossible to recover the symmetric key.

#### Justification of secrete key security

The security of the process relies on the properties of the XOR operation and the fact that the shared key is known only to the legitimate parties (Alice and Bob).

The XOR operation has the property that if you know the result $C = A \oplus B$ and one of the operands (either $A$), one can uniquely determine the other operand ($B = C \oplus A$). However, if you only know $C$ without knowing either $A$ or $B$, it is computationally infeasible to determine either $A$ or $B$ without additional information.

In the proposed setup, the shared key $K$ is known only to Alice and Bob, and it is never shared publicly. Even if an attacker (Eve) intercepts the merged output, without knowledge of the shared key $K$, the receiver cannot recover the symmetric key $\alpha$.

#### Mathematical proof of security

##### Theorem 1

Prove that an attacker who intercepts the merged output $M = \alpha \oplus K$ cannot recover $\alpha$ without knowing $K$.

- **Given:**
- Symmetric key: $$\alpha$$
- Shared key: $$K$$
- Merged output: $$M = \alpha \oplus K$$
- **To prove:** Knowing $M$ alone does not allow an attacker to recover $\alpha$.

**Case 1:** Eve has only the merged output $M = \alpha \oplus K$. To recover $\alpha$, Eve needs to compute:$\alpha = M \oplus K$.

**Challenge for Eve: ** Eve knows $M$, but $M$ depends on both $\alpha$ and $K$. $K$ is unknown to Eve. Therefore, the XOR operation is such that every possible key $K$ could correspond to a different symmetric key $\alpha$. This means that without knowing $K$, Eve cannot uniquely determine $\alpha$.

**Case 2: Randomness of** $K$: Assume $K$ is a random string of bits, generated independently of $\alpha$. Since $K$ is random and known only to Alice and Bob, the merged output $M$ can be any random binary sequence of the same length as $\alpha$. Therefore, for every possible value of $M$, there is an equally likely $K$ that could correspond to any possible $\alpha$. Thus, from $M$ alone, the probability distribution of possible values of $\alpha$ is uniform over all possible values, providing no information about $\alpha$.

**Case 3: Conditional Entropy: ** The conditional entropy $H(\alpha | M)$ represents the uncertainty about $\alpha$ given $M$. Since $K$ is random and unknown to Eve, the conditional entropy remains high: $H(\alpha | M) = H(\alpha )$. This means that knowing $M$ does not reduce the uncertainty about $\alpha$.

#### Image encryption

In the proposed encryption scheme following major components are used to provide robust security to the digital images.

- Bit-plane extraction-based cryptography
- Random numbers generated using chaos
- Confusion phase: Multiple scrambling operations
- Diffusion phase: XOR operation, Affine transformations with nonlinear functions, discrete cosine transform (DCT), wavelet transform with Random Phase Modulation, bilinear transformation with complex weights, and nonlinear polynomial mapping

#### Bit-plane extraction based cryptography

Let $I$ be the input color image, which can be represented as a 3D matrix $I(x, y, c)$ where $x$ and $y$ are the spatial coordinates, and $c$ denotes the color channel (Red, Green, Blue) as given in Equation 4.

4 $$\begin{aligned} I(x, y, c) = {\left\{ \begin{array}{ll} R(x, y) & \text {if } c = 1 \\ G(x, y) & \text {if } c = 2 \\ B(x, y) & \text {if } c = 3 \end{array}\right. } \end{aligned}$$

For each color component $C(x, y) \in \{R(x, y), G(x, y), B(x, y)\}$, extract the $k$-th bit-plane $P_{C,k}(x, y)$ using Equation 5.

5 $$\begin{aligned} P_{C,k}(x, y) = \left\lfloor \frac{C(x, y)}{2^k} \right\rfloor \mod 2 \end{aligned}$$

where $k$ ranges from 0 to 7, representing each bit-plane. Apply encryption functions as given in Equations 6-10 to each bit-plane extracted from the R, G, and B components. Suppose, there are $n$ rounds of encryption with a round key $K_i$ for each round $i$.

Now, use a substitution function $S$ (S-box) for non-linear substitution suing Equation 6. The S-box used in this research are given in^41–43^.

6 $$\begin{aligned} S(P_{C,k}(x, y)) = \text {S-box}(P_{C,k}(x, y)) \end{aligned}$$

The permutation layer, which scrambles the bits, and the key mixing, which XORs the result with the round key, are applied according to Equation 7.

7 $$\begin{aligned} {\left\{ \begin{array}{ll} P_{C,k}'(x, y) = P(S(P_{C,k}(x, y)))\\ P_{C,k}''(x, y) = P_{C,k}'(x, y) \oplus K_i \end{array}\right. } \end{aligned}$$

Repeat substitution, permutation, and key mixing for $n$ rounds. Let $P_{C,k}^n(x, y)$ be the pre-encrypted bit-plane after $n$ rounds as given in Equation 8.

8 $$\begin{aligned} P_{C,k}^n(x, y) = P_n \left( \text {S}_n \left( \bigoplus _{i=1}^{n} (P_{C,k}^{i-1}(x, y) \oplus K_i) \right) \right) \end{aligned}$$

where $\text {S}_n$ and $P_n$ represent the substitution and permutation operations in the $n$-th round.

Combine the encrypted bit-planes to create the pre-ciphertext image by reconstructing each pixel value for each color component from the encrypted bit-planes using Equation 9.

9 $$\begin{aligned} C_{\text {enc}}(x, y) = \sum _{k=0}^{7} P_{C,k}^n(x, y) \cdot 2^k \end{aligned}$$

where $C \in \{R, G, B\}$ denotes the color component. Combine the encrypted color components to form the pre-encrypted image $I_{\text {P-enc}}$ as given in Equation 10. The process to generate $P-enc$ is given in Algorithm 1.

10 $$\begin{aligned} P_{\text {enc}}(x, y, c) = {\left\{ \begin{array}{ll} R_{\text {enc}}(x, y) & \text {if } c = 1 \\ G_{\text {enc}}(x, y) & \text {if } c = 2 \\ B_{\text {enc}}(x, y) & \text {if } c = 3 \end{array}\right. } \end{aligned}$$

#### Random numbers generation using chaos

To generate a random image using a chaotic map and convert it to a 2D image, the following steps are followed:

- **Choose initial values and control parameters:** Select initial values $x_0$ and control parameters $a$ for the chaotic map.
- Iterate Equations 1, and 2 for $M \times N$ times to generate a sequence of values.
- **Scale the Values: ** Since the chaotic map produces values in the range $(0, 1)$, multiply each value by a large integer $L$ to amplify it as given in Equation 11.
  11 $$\begin{aligned} y_n = x_n \cdot L \end{aligned}$$
- Truncate the decimal part of $y_n$ to convert it into an integer value.
- **Adjust to the desired range [0, 255]: **Take the modulo 256 of each value to ensure it fits within the 8-bit range according to Equation 12.
  12 $$\begin{aligned} z_n = \text {mod}(y_n, 256) \end{aligned}$$
- **Generate the image: ** Reshape the 1D sequence of values into a 2D matrix with size $M \times N$, where $M \times N$ is the total number of color components in the image.

#### Confusion phase

In the confusion phase of the proposed encryption framework, multiple image shuffling techniques such as pixel shuffling, pixel value permutation, rotation and flipping, and scrambling by image transformation are applied to $P_{enc}$.

**Algorithm 1:**
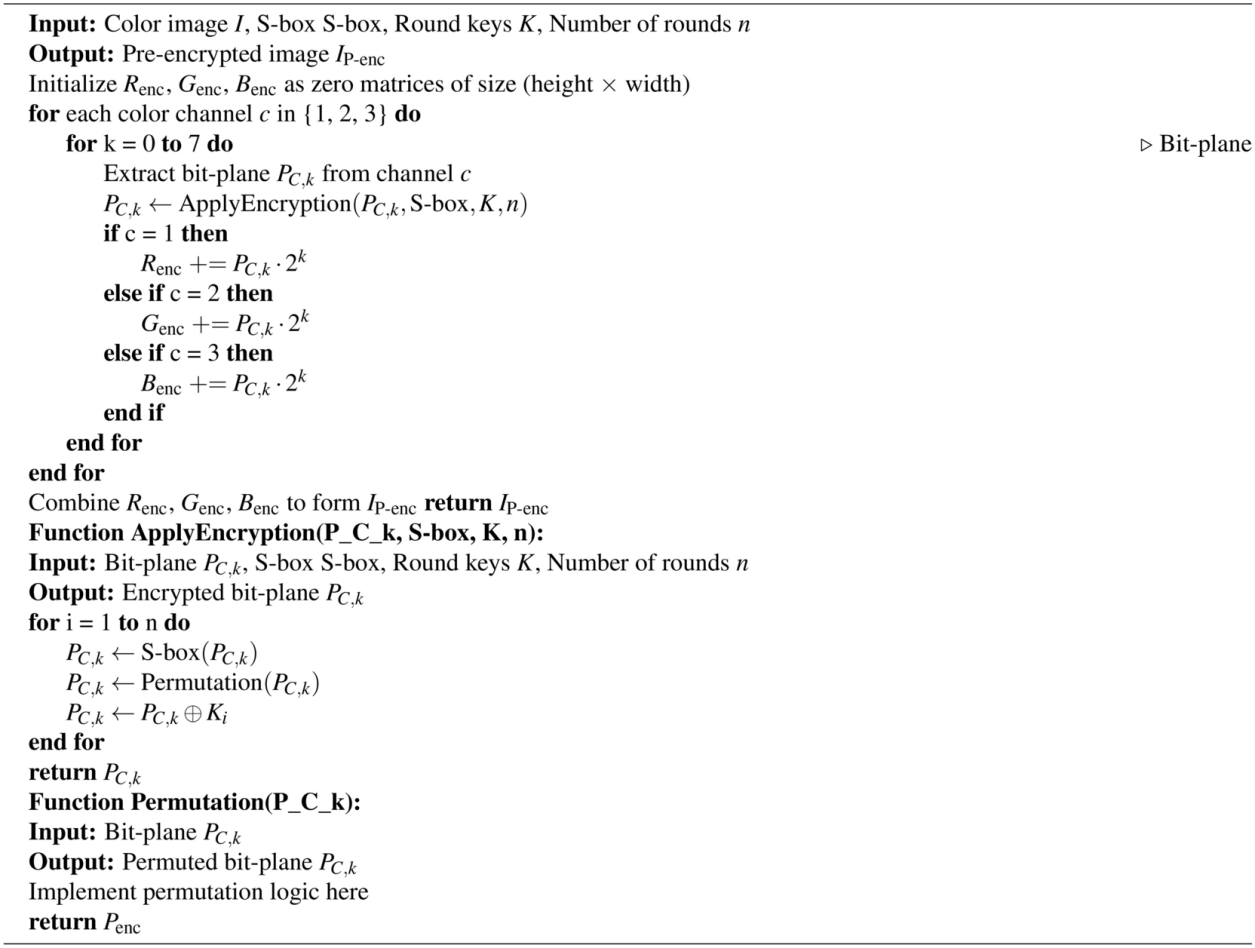
Bit-plane extraction based permutation.

#### Pixel shuffling

It involves rearranging the pixels of an image according to some permutation rules. The primary goal is to disrupt the spatial structure of the image without altering the pixel values. In the proposed encryption frame work, given an image $P_{enc}$ of size $M \times N$, let each pixel be denoted as $P_{enc}(i, j)$, where $i \in \{1, 2, \ldots , M\}$ and $j \in \{1, 2, \ldots , N\}$. The two types of pixel shuffling such as row and column scrambling are used. For the row scrambling, a permutation function is defined as $\pi _r: \{1, 2, \ldots , M\} \rightarrow \{1, 2, \ldots , M\}$ that randomly shuffles the rows of the $P_{enc}$. The new image $P_{shuffled\_rows}$ after row shuffling is given by Equation 13.

13 $$\begin{aligned} \begin{aligned} P_{shuffled\_rows}(\pi _r(i), j) = P_{enc}(i, j), \\ \forall i \in \{1, 2, \ldots , M\}, \forall j \in \{1, 2, \ldots , N\} \end{aligned} \end{aligned}$$

For the column scrambling, a permutation function is defined as $\pi _c: \{1, 2, \ldots , N\} \rightarrow \{1, 2, \ldots , N\}$ that randomly shuffles the columns of $P_{shuffled\_rows}(\pi _r(i), j)$. The new image $P_{shuffled}$ after both row and column shuffling is given by Equation 14.

14 $$\begin{aligned} \begin{aligned} P_{shuffled}(\pi _r(i), \pi _c(j)) = P_{enc}(i, j), \\ \forall i \in \{1, 2, \ldots , M\}, \forall j \in \{1, 2, \ldots , N\} \end{aligned} \end{aligned}$$

#### Pixel value permutation

It changes the order of pixel values in the image without changing their spatial locations. This technique disrupts the color distribution while maintaining the structural content. In this step, there are also two techniques of permutation such as flattening the image, and a permutation of pixel values is applied. First, flatten the image ($P_{shuffled}(\pi _r(i), \pi _c(j))$) into a one-dimensional vector $\textbf{p} = [p_1, p_2, \ldots , p_{MN}]$, where $p_k = P_{enc}(i_k, j_k)$ for $k = 1, 2, \ldots , MN$. Whereas, in the permutation of pixel values, first define a permutation function $\pi _v: \{1, 2, \ldots , MN\} \rightarrow \{1, 2, \ldots , MN\}$ that rearranges the pixel values. The new pixel vector $\mathbf {p'}$ after permutation is $\mathbf {p'}(\pi _v(k)) = \textbf{p}(k), \quad \forall k \in \{1, 2, \ldots , MN\}$. After that, reshape the permuted vector $\mathbf {p'}$ back to the original image dimensions $M \times N$ to obtain the permuted image $P_{perm}$.

#### Rotation and flipping

 To rotate an image$P_{shuffled}(\pi _r(i), \pi _c(j))$ by$\theta$ degrees around its center, each pixel$P_{enc}(i, j)$ is mapped to a new location$(i', j')$ using the rotation matrix which is given in Equation15.

15 $$\begin{aligned} \begin{bmatrix} i' \\ j' \end{bmatrix} = \begin{bmatrix} \cos \theta & -\sin \theta \\ \sin \theta & \cos \theta \end{bmatrix} \begin{bmatrix} i - i_c \\ j - j_c \end{bmatrix} + \begin{bmatrix} i_c \\ j_c \end{bmatrix} \end{aligned}$$

where$(i_c, j_c)$ is the center of the image, calculated as:$i_c = \frac{M + 1}{2}, \quad j_c = \frac{N + 1}{2}$.

To flip the image, both horizontal and vertical flipping techniques are employed according to the Equations 16, and 17, respectively.

16 $$\begin{aligned} P_{flip\_horizontal}(i, j)= & P_{perm}(i, N - j + 1), \nonumber \\ & \forall i \in \{1, 2, \ldots , M\}, \forall j \in \{1, 2, \ldots , N\} \end{aligned}$$

17 $$\begin{aligned} P_{flip\_vertical}(i, j)= & P_{flip\_horizontal} (M - i + 1, j), \nonumber \\ & \forall i \in \{1, 2, \ldots , M\}, \forall j \in \{1, 2, \ldots , N\} \end{aligned}$$

Algorithm 2 provides the pseudocode used to generate the confusion depicted in the image.

**Algorithm 2:**
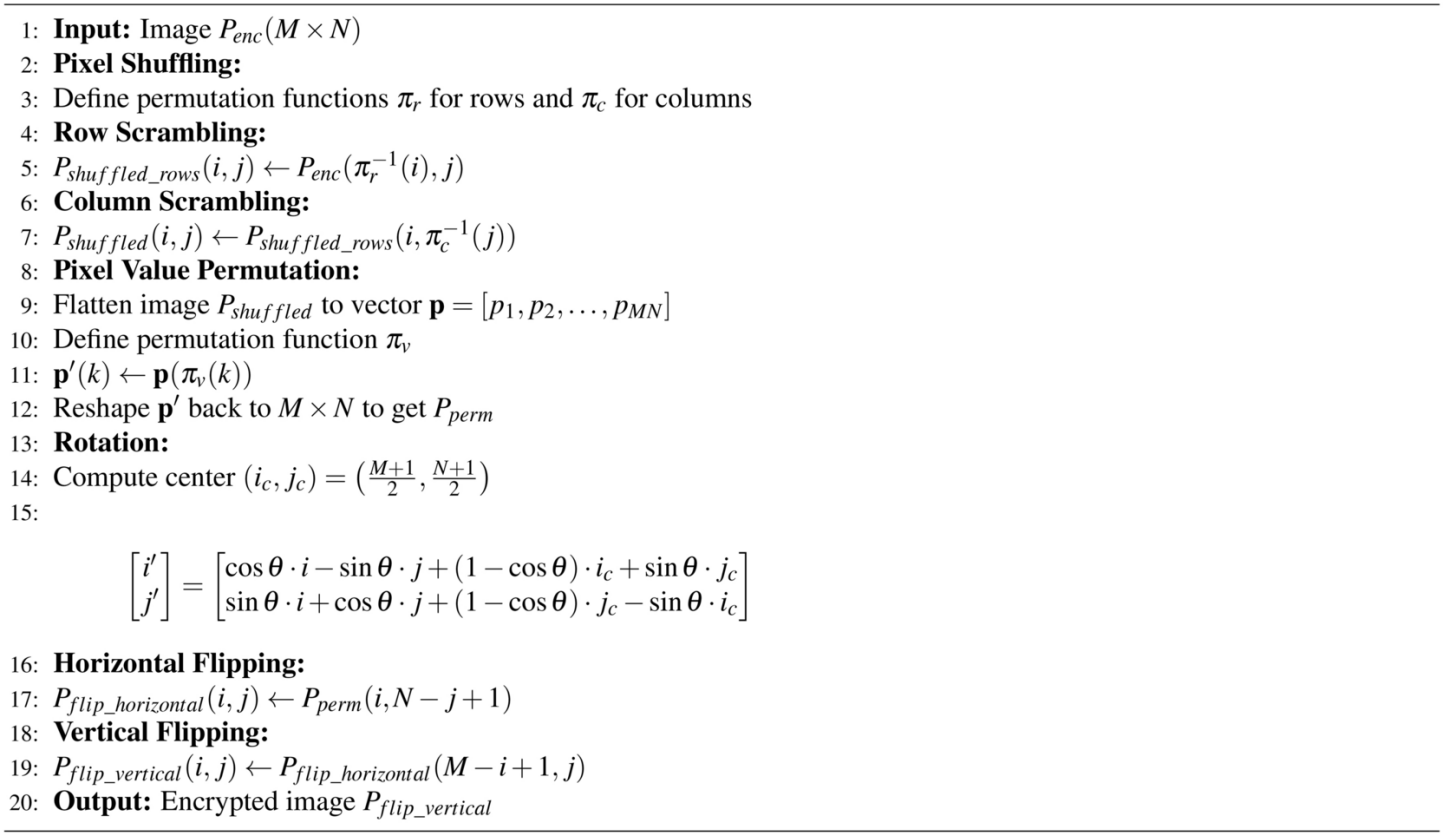
Confusion phase.

#### Diffusion phase

The objective of this phase is to disperse the influence of each pixel in $I_s$ throughout the entire image for enhancing the overall security. In this research, this is achieved using a different complex mathematical operation. The detail of such operations is provided in the next subsections.

#### Affine transformation with nonlinear functions

Affine transformation is integrated with nonlinear functions to enhance diffusion. There are two major transformations in this process: (i) Affine transformation and (ii) nonlinear function application. Mathematical representations of these transformations are given in Equations 18, and 19, respectively.

18 $$\begin{aligned} \textbf{A}(i, j)= & a \cdot \mathbf { P_{flip\_vertical}}(i, j) + b \end{aligned}$$

19 $$\begin{aligned} \textbf{A}(x, y)= & \left\lfloor \left( a \cdot \mathbf { P_{flip\_vertical}}(x, y) + b\right) \cdot \left[ \sin \left( \frac{(x+y) \cdot \pi }{N \cdot M}\right) \right. \right. \nonumber \\ & \quad \left. \left. + \left( \frac{(x-y)^2}{\sqrt{N \cdot M}}\right) ^p \right] \right\rfloor \end{aligned}$$

Where $a$ and $b$ are constants, and $p$ is a polynomial degree.

#### Discrete Cosine Transform (DCT) with complex modulation

Apply the 2D DCT to the $\textbf{A}(x, y)$ using Equation 20.

20 $$\begin{aligned} \textbf{D}(x, y) = \sum _{x=0}^{N-1} \sum _{y=0}^{M-1} \textbf{A}(x, y) \cdot \cos \left[ \frac{\pi (2i + 1)}{2N}\right] \cdot \cos \left[ \frac{\pi (2j + 1)}{2M}\right] \end{aligned}$$

After applying 2d DCT, use a complex modulation function to the DCT coefficients to get a second pre-ciphertext image ($\mathbf {D'}(x, y)$) using Equation 21.

21 $$\begin{aligned} \mathbf {D'}(x, y) = \textbf{D}(x, y) \cdot \left( \exp \left[ \frac{ \pi }{N + M}\right] \cdot \sin \left( \frac{\pi ^2 }{N^2 \cdot M^2}\right) \right) \end{aligned}$$

Now, apply the inverse 2D DCT using Equation 22 to obtain the third ciphertext image ($\mathbf {D''}(u, v)$).

22 $$\begin{aligned} \begin{aligned} \mathbf {D''}(x, y)&= \frac{2}{NM} \sum _{x=0}^{N-1} \sum _{y=0}^{M-1} \mathbf {D'}(x, y)\\&\cdot \cos \left[ \frac{\pi (2x + 1)}{2N}\right] \cdot \cos \left[ \frac{\pi (2y + 1)}{2M}\right] \end{aligned} \end{aligned}$$

#### Wavelet transform with random phase modulation

In this research, the wavelet transform is used to decompose $\mathbf {D''}(x, y)$ into different frequency sub-bands (LL, LH, HL, and HH) which consist of both high- and low-frequency components. The 2D wavelet transform is implemented using Equation 23.

23 $$\begin{aligned} \textbf{W}(j, k, a, b) = \sum _{x, y} \mathbf {D''}(x, y) \cdot \psi _{j, k}(x - a, y - b) \end{aligned}$$

Where $\textbf{W}(j, k, a, b)$, and $\psi _{j, k}(x - a, y - b)$ are the wavelet coefficients, and wavelet function, respectively. The wavelet function is shifted by $(a, b)$ and scaled according to indices $j$ and $k$. The random phase modulation is applied to the wavelet coefficients to for the modification of phase information of the image components using Equation 24.

24 $$\begin{aligned} \begin{aligned} \mathbf {W'}(j, k, a, b)&= \textbf{W}(j, k, a, b) \cdot \Bigg [\exp \left( \textbf{i} \cdot \frac{\pi \cdot (a + b)}{M \cdot N}\right) \\&\cdot \left( \sin \left( \frac{2 \pi \cdot j \cdot k}{N}\right) + \frac{a}{b} \cdot \cos \left( \frac{\pi \cdot (a^2 + b^2)}{M \cdot N}\right) \right) \Bigg ] \end{aligned} \end{aligned}$$

Where $a$ and $b$ are constants. The inverse wavelet transform is applied using Equation 25.

25 $$\begin{aligned} \textbf{C}(x, y) = \sum _{j, k} \sum _{a, b} \mathbf {W'}(j, k, a, b) \cdot \psi _{j, k}(x - a, y - b) \end{aligned}$$

#### Bilinear transformation

The purpose of bilinear transformations is to create non-linear relationships between image pixels in a way that is not easily reversible without exact replica of the secrete keys and the knowledge of the transformation parameters. Furthermore, adding a bilinear transformation to the encryption process enhances the security of the enciphered images. The bilinear transformation for $\textbf{C}(x, y)$ is given in Equation 26.

26 $$\begin{aligned} \textbf{B}(i, j) = \sum _{x=0}^{N-1} \sum _{y=0}^{M-1} \textbf{C}(x, y) \cdot \left[ \frac{(i+k) \cdot (j+l)}{N \cdot M} \right] \end{aligned}$$

To increase further complexity, a complex weighting function is applied to the transformed pixel values using Equation 27.

27 $$\begin{aligned} \begin{aligned} \mathbf {C'}(i, j)&= \left\lfloor \textbf{B}(i, j) \cdot \left[ \frac{1}{1 + \exp \left( -\frac{(i+j)}{\sqrt{N \cdot M}}\right) }\right. \right. \\&\quad \left. \left. + \cos \left( \frac{\pi \cdot (i^2 + j^2)}{N \cdot M}\right) \right] \right\rfloor \end{aligned} \end{aligned}$$

#### Nonlinear polynomial mapping

In the last step of the proposed encryption framework, a nonlinear polynomial mapping is applied to change the pixel values based on higher-order polynomials. This introduces non-linearity and makes the transformation difficult to reverse without knowing the exact polynomial used. A nonlinear polynomial mapping is applied to each pixel value to produce the final ciphertext image ($C_f(I,j)$) using Equation 28.

28 $$\begin{aligned} \begin{aligned} \mathbf {C_f}(i, j)&= \left\lfloor \left( C(i, j)^3 + \frac{\pi \cdot (i \cdot j)}{N + M}\right) \cdot \left[ \exp \left( \frac{i^2 + j^2}{N \cdot M}\right) \right. \right. \\&\quad \left. \left. + \sin \left( \frac{2 \pi \cdot i \cdot j}{N \cdot M}\right) \right] \right\rfloor \end{aligned} \end{aligned}$$

**Fig. 4:**
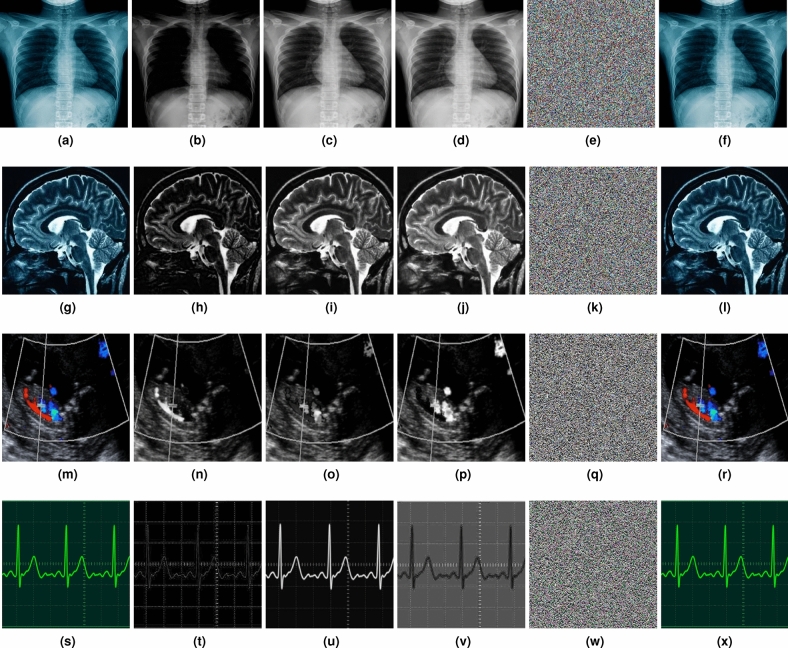
(**a**,**f**,**k**, **p**) Plaintext images, (**b**-**d**, **g**-**i**, **l**-**n**, **q**-**s**) their corresponding R, G, B components, and (**e**, **j**, **o**, **k**) corresponding decrypted versions.

The pseudocode of the proposed diffusion phase is given in Algorithm 3. The entire encryption framework provides a significantly robust non-linear relationship between the original, and the final ciphertext image. The generated ciphertext images are displayed in Figure 4, where all plaintext information is entirely concealed, and the original image is unrecognizable. This demonstrates the effectiveness of the proposed framework in encrypting the plaintext image by disrupting pixel correlations.

## Experimental results and analysis

To test and analyze the proposed encryption framework for securing digital images, four test images are used: X-ray, Tumor, Ultrasound, and Electrocardiogram (ECG) signal images. The entire encryption framework is implemented in MATLAB 2018 on a system with the following hardware specifications: 8 GB RAM, 512 GB SSD, $11^{th}$ generation Intel Core i5 processor at 2.4 GHz, running Windows 11. The effectiveness of the encryption framework is assessed through various statistical security analyses, including entropy, histogram, lossless analysis, and key sensitivity. The statistical values for the encrypted images are averaged across the R, G, and B components. The framework is also tested against multiple cyberattacks including brute force, noise, and cropping attacks to show its resilience to these threats.

### Algorithm test

The proposed encryption framework is tested on different plaintext color images including X-ray and tumor images with a resolution of $256 \times 256$, as well as ultrasound and ECG signal images with a resolution of $512 \times 512$ having varying sizes such as $256 \times 256$, and $512 \times 512$. The quality of the encrypted images is then assessed visually. As shown in Figure 4, the plaintext information is completely concealed within the ciphertext images, with no visible patterns of the original data. This shows that the proposed encryption framework is effective in protecting plaintext information.

### Entropy

Entropy measures the randomness in an image, with the ideal value varying based on the bit depth of the image. The maximum entropy value for an 8-bit image is 8, while for a binary (2-bit) image, it is 2. Mathematically, it can be calculated using Equation 29.

29 $$\begin{aligned} E_I = \sum ^{M}_{L=0} p(i_l)log_bp(i_l) \end{aligned}$$

Where, $E_I$ is the entropy of any image. $log_b(p(i_l))$ is the logarithm of the probability $p(t_k)$ with a base $b$. The base $b$ depends on the entropy calculation. For example, if $b = 2$, it is a binary logarithm, which is commonly used when dealing with bits. $\sum ^{N}_{K=0}$ is the summation symbol which indicates that the sum is calculated over all possible pixel values $L$ in the image ranges form $0$ to $M$. For robust encryption, it is essential that the entropy value is as high as possible. A higher entropy value indicates greater randomness in an image, which contributes to stronger encryption. The relationship between high entropy and robust encryption is expressed in Equation 30.

30 $$\begin{aligned} \text {Entropy} \propto \text {Strong Encryption} \end{aligned}$$

Table 3 presents entropy values for both the proposed and existing methods. The results show that the entropy values of the encrypted images from the proposed framework are closer to 8, indicating a higher level of randomness in the ciphertext compared to existing methods.

**Table 3:** Entropy analysis.

Image size	Images	^32^	^28^	^44^	^45^	^46^	Proposed
$256 \times 256 \times 3$	Xray	7.9967	7.9919	7.9936	7.9955	7.9949	7.9993
	Tumor	7.9946	7.9966	7.9919	7.9977	7.9991	7.9994
$512 \times 512 \times 3$	Ultrasound	7.9961	7.9979	7.9987	7.9949	7.9966	7.9992
	ECG signal	7.9916	7.9910	7.9933	7.9969	7.9982	7.9991

**Algorithm 3:**
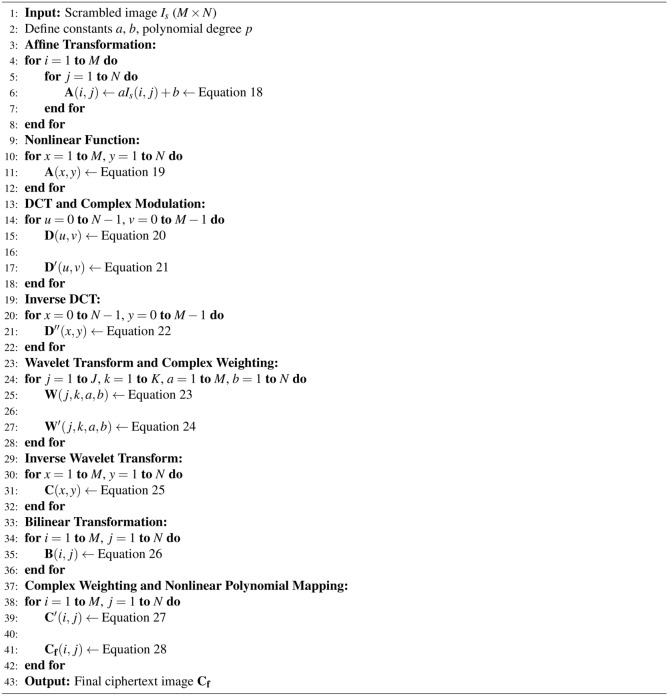
Diffusion phase.

**Fig. 5:**
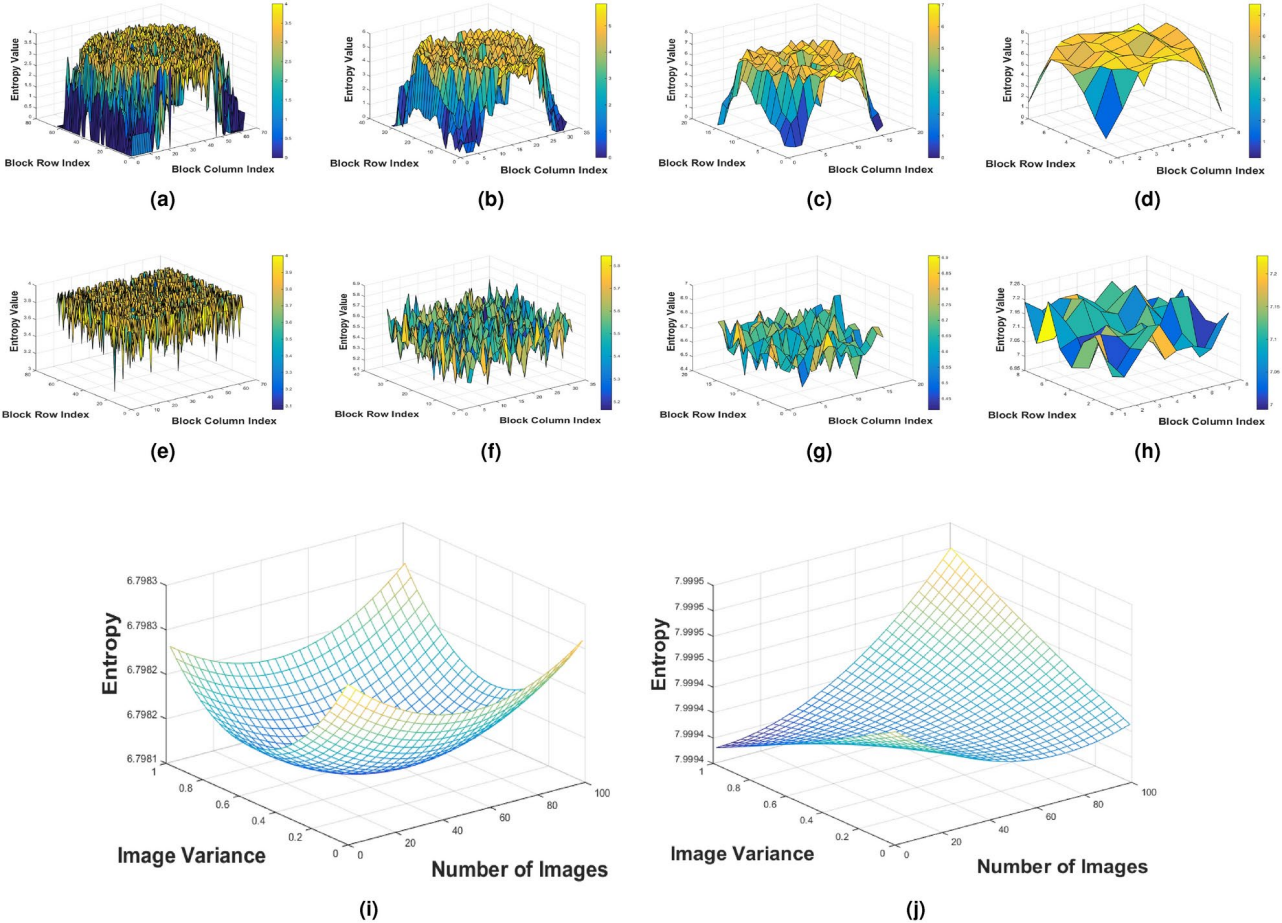
Polynomial surface fit plots for entropy values of 100 original and corresponding encrypted images.

In addition to the global entropy calculation, the encrypted and plaintext images are divided into blocks of various sizes containing 16, 64, 256, and 1024 pixels in each block. The 3D entropy distribution plots for these blocks are displayed in Figure 5. Even with a block size of $4 \times 4$, the entropy remains above approximately 3.58, indicating that no pattern of even 16 pixels repeats in the encrypted image, with each pixel appearing random. This results in a high global entropy, as shown in Table 3. In contrast, the 3D block-wise entropy distribution for the plaintext image in Figure 5(a-d) shows entropy dropping to zero for 4x4 and 8x8 blocks, revealing smooth patterns in the plaintext image. Furthermore, the global entropy for 100 plaintext and corresponding ciphertext images is displayed in the polynomial surface fit plots in Figure 5 (i, j). It can be observed that the global entropy of the plaintext images falls within the range of approximately [6.7981, 6.79983], while for the corresponding ciphertext images, it ranges from roughly [7.9994, 7.9995].

### Correlation analysis

Correlation evaluates how adjacent pixels relate to each other in different orientations. In general, plaintext images exhibit high pixel correlation because of the similarity between neighboring pixels. However, in image encryption, it is crucial to break this pixel correlation to protect the plaintext information securely. Reduced correlation indicates a more robust encryption, as it signifies that the encryption process has effectively broken the inherent relationships between pixels, enhancing the security of the image. The pixel correlation coefficients for horizontally (H), vertically (V), and diagonally (D) adjacent pixels are calculated using Equations 31, 32, and 33 to assess the effectiveness of the proposed encryption framework.

31 $$\begin{aligned} \text {Corr}_{\text {horizontal}}= & \frac{\sum _{j=1}^{W-1} (I(i, j) - \bar{I}_i) (I(i, j+1) - \bar{I}_i)}{(W-1) \sigma _i^2} \end{aligned}$$

32 $$\begin{aligned} \text {Corr}_{\text {vertical}}= & \frac{\sum _{i=1}^{H-1} (I(i, j) - \bar{I}_j) (I(i+1, j) - \bar{I}_j)}{(H-1) \sigma _j^2} \end{aligned}$$

33 $$\begin{aligned} \text {Corr}_{\text {diagonal}}= & \frac{\sum _{i=1}^{H-1} \sum _{j=1}^{W-1} (I(i, j) - \bar{I}) (I(i+1, j+1) - \bar{I})}{(N-1) \sigma ^2} \end{aligned}$$

In Equations 31-33, $I(i, j)$ represents the pixel value at row $i$ and column $j$. $W$ and $H$ denote the width and height of the image, respectively; $\bar{I}_i$ is the mean pixel value for row $i$ or column $j$; and $\sigma _i^2$ is the variance of pixel values in row $i$. Table 4 displays various correlation values, showing that the ciphertext images exhibit minimal correlations in all directions compared to existing encryption methods, with coefficients approaching zero. This indicates that the proposed encryption method is more effective at randomizing pixel values and disrupting the patterns present in the plaintext information.

**Table 4:** Correlation analysis.

Image size	Images	Directions	^32^	^28^	^44^	^45^	^46^	Proposed
$256 \times 256 \times 3$	Xray	H	0.0023	0.0015	0.0014	0.0016	0.0028	0.0001
		V	0.0016	0.0019	0.0022	0.0019	0.0029	0.0002
		D	0.0011	0.0031	0.0026	−0.0016	0.0019	−0.0003
	Tumor	H	0.0014	0.0013	−0.0023	−0.0015	0.0014	−0.0013
		V	0.0011	0.0021	−0.0016	0.0031	−0.0020	0.0001
		D	0.0011	0.0013	−0.0011	0.0018	−0.0011	−0.0007
$512 \times 512 \times 3$	Ultrsound	H	0.0021	−0.0022	−0.0013	0.0022	−0.0016	−0.0002
		V	0.0011	0.0019	−0.0017	0.0031	0.0010	0.0003
		D	0.0016	0.0021	−0.0033	0.0017	0.0033	0.0006
	ECG signal	H	0.0023	−0.0014	−0.0013	−0.0017	0.0032	−0.0013
		V	0.0016	−0.0011	0.0031	0.0008	0.0031	0.0005
		D	0.0034	0.0064	0.0046	0.0044	−0.001	−0.0003

**Fig. 6:**
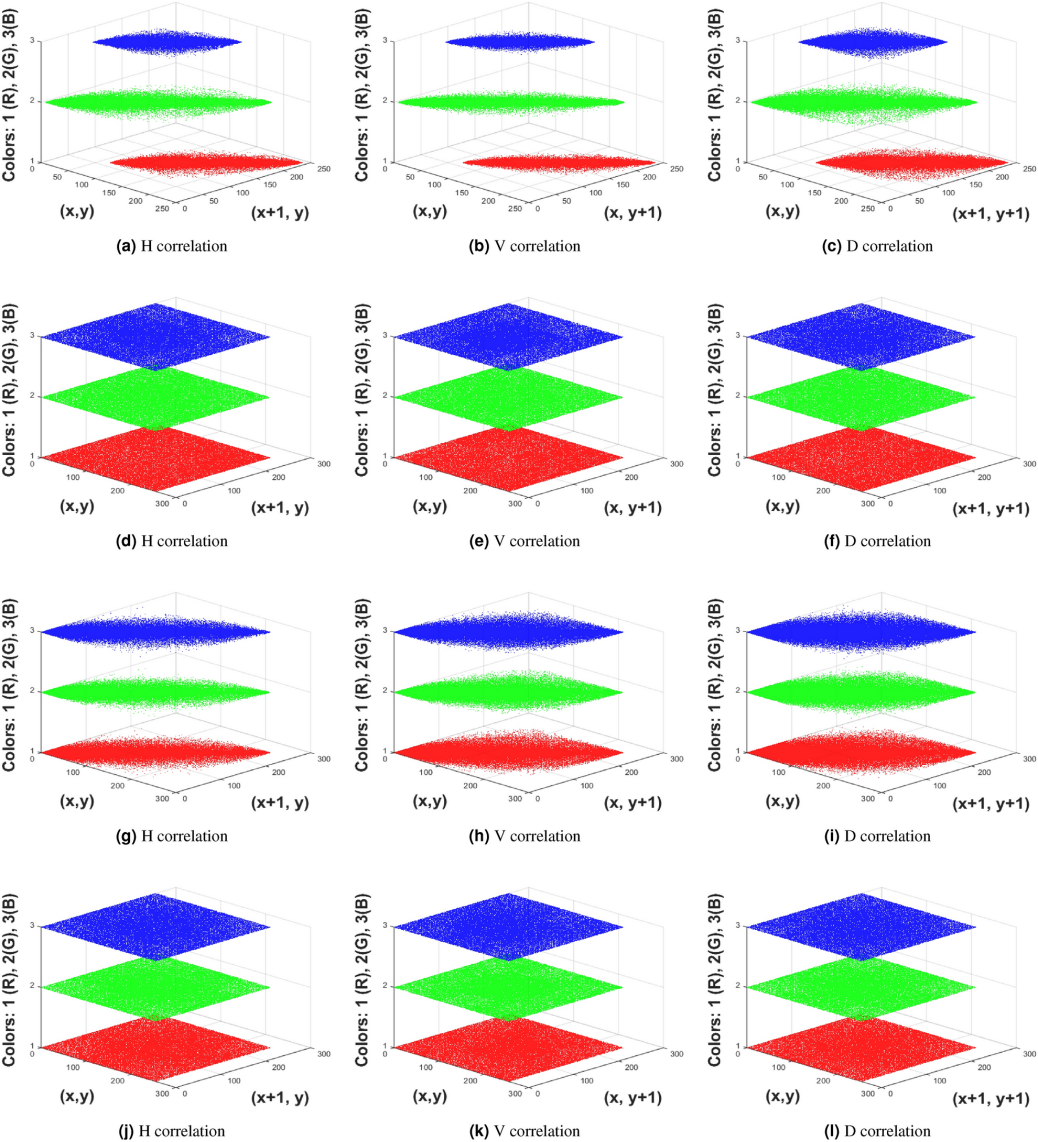
Correlation in multi directions (**a**-**c**) Correlation in Tumor image, (**d**-**f**) Correlation in encrypted Tumor image, (**g**-**i**) (**a**-**c**) Correlation in Xray image, (**j**-**l**) Correlation in encrypted Xray image.

A visual analysis using scatter plots shows the statistical correlation analysis. Figure 6 compares the pixel correlations of the plaintext and ciphertext images. The closely clustered points in Figures (a-c, g-i) indicate a high correlation among plaintext pixels, while the more scattered points in Figures (d-f, j-l) demonstrate very little correlation in the ciphertext pixels.

### Key sensitivity analysis

For any encryption framework, it is crucial that the secret keys used are highly sensitive. Sensitivity to secret keys means that even a tiny change in the keys makes it impossible to successfully decrypt the plaintext information. In this work, a total of fifteen secret keys ($$\varpi _1, \varpi _2, \cdots , \varpi _6,$$ $$\sigma _1, \rho _1, \beta _1$$,$$\sigma _2, \rho _2, \beta _2, \Xi , \xi , u, k, p$$) are used. To perform the key sensitivity analysis, a minute change of $$\Delta = 10^{-15}$$ is introduced to each secret key. The modified keys are $$\varpi '_1 = \varpi _1 + \Delta , \varpi '_2 = \varpi _2 + \Delta , \ldots , \varpi '_6 =$$ $$\varpi _6 + \Delta , \sigma '_1 = \sigma _1 + \Delta , \rho '_1 = \rho _1 + \Delta , \beta '_1 = \beta _1 + \Delta , \sigma '_2 = \sigma _2 + \Delta , \rho '_2 = \rho _2 + \Delta ,$$$$\beta '_2 = \beta _2 + \Delta , \Xi ' = \Xi + \Delta , \xi ' = \xi + \Delta , u' = u + \Delta , k' = k + \Delta , p' = p + \Delta$$. These modified keys are then used to decrypt the plaintext image from the ciphertext image.

**Fig. 7:**
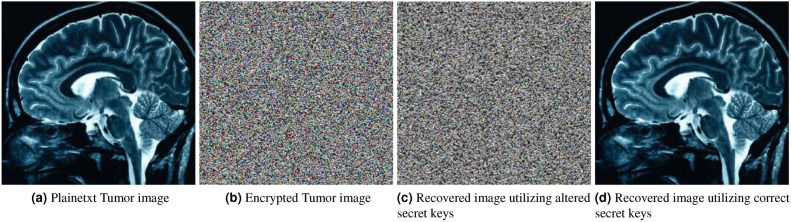
Key sensitivity analysis.

The resulting decrypted images, which are recovered using the modified secret keys, are shown in Figure 7. It can be observed in Figure 7(c) that the decrypted images contain no recognizable information from the original plaintext image, which indicates that even a tiny modification to the secret keys renders decryption completely ineffective. This demonstrates that the secret keys used in the proposed encryption framework are highly sensitive. To successfully decrypt the plaintext information, the exact keys must be used. Figure 7 (d) shows the decrypted image using the correct keys, where it is evident that the image is successfully recovered.

### Key space analysis

Keyspace analysis refers to the size of the secret keys used in an encryption framework and is also related to brute force attacks. In a brute force attack, an adversary attempts every possible combination of secret keys to breach the encryption. To determine the size of the secret keys in the proposed encryption framework, first assess key sensitivity as explained in Section 5.4. In this proposed framework, the sensitivity of each key is at least $10^{-15}$, meaning the size of each secret key is $10^{+15}$. Therefore, the total key space for the fifteen keys is $10^{15 \times 15}$, which is approximately $2^{747.43}$. According to Alvarez’s^47^criteria for key space, any encryption algorithm with a key space equal to or greater than $2^{100}$ is considered secure against brute force attacks.

### Noise and cropping attack analysis

To make decryption fail, attackers often attempt to modify the ciphertext image by adding external noise to it or cropping a portion of the ciphertext image.

In the ciphertext image, 10% salt and 10% pepper noise are added randomly to alter each pixel’s value. To evaluate the proposed encryption framework, first, random noise is added to the ciphertext image pixels. For each pixel at position $(i, j)$ in the image $I$, the noisy image $I'$ will be calculated using Equation 34.

34 $$\begin{aligned} I'(i, j) = {\left\{ \begin{array}{ll} 0 & \text {with probability } 0.1 \quad (\text {pepper noise}) \\ 255 & \text {with probability } 0.1 \quad (\text {salt noise}) \\ I(i, j) & \text {with probability } 1 - 0.2 = 0.8 \quad (\text {no change}) \end{array}\right. } \end{aligned}$$

**Fig. 8:**
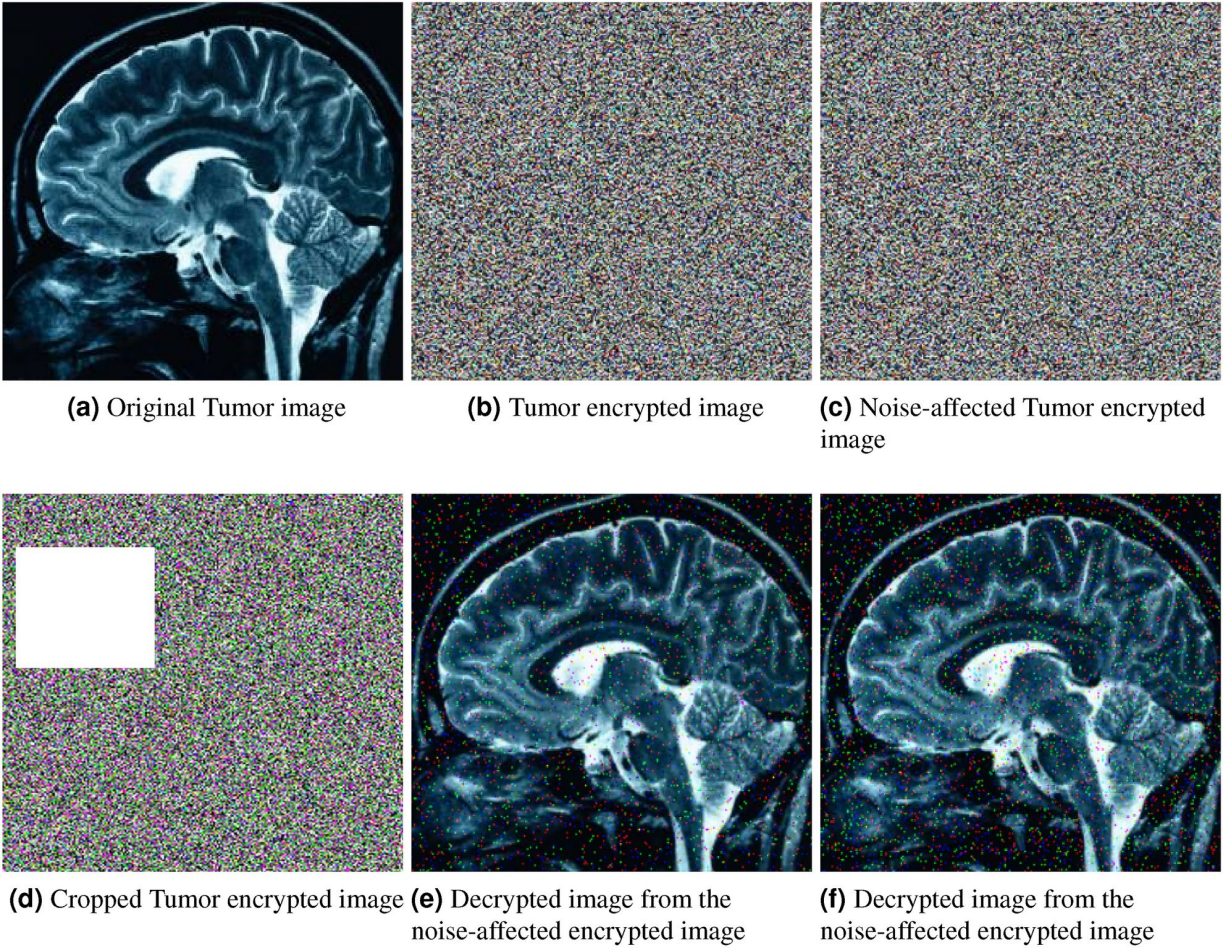
Noise and cropping attack analysis.

After adding noise to the ciphertext image pixels, the plaintext image is recovered from the noisy version of the ciphertext. The visualized results are displayed in Figure 8. The decrypted image, shown in Figure 8(e), demonstrates that the information in the plaintext image is still visible, although some noise remains in the recovered image. This indicates that while the exact pixel values are not fully restored, the content of the plaintext image is still easily recognizable. This result shows that the proposed encryption framework is effective in resisting noise attacks.

To perform a cropping attack analysis on the encryption framework, 20% portion of the ciphertext image is cropped and then attempt to decrypt the plaintext image from this cropped version. The decrypted image is shown in Figure 8(f). Based on your cropping attack analysis, the results indicate that the proposed encryption framework is effective at resisting cropping attacks. Despite removing 20% of the ciphertext image, the decryption process is able to produce an output where the plaintext image remains largely recognizable. This suggests that the encryption framework is resilient to partial data loss, as significant portions of the original content are still recoverable from the cropped ciphertext. The ability of the framework to maintain the integrity of the plaintext image under such conditions shows its robustness and effectiveness in handling attacks that involve manipulating or removing portions of the ciphertext.

**Fig. 9:**
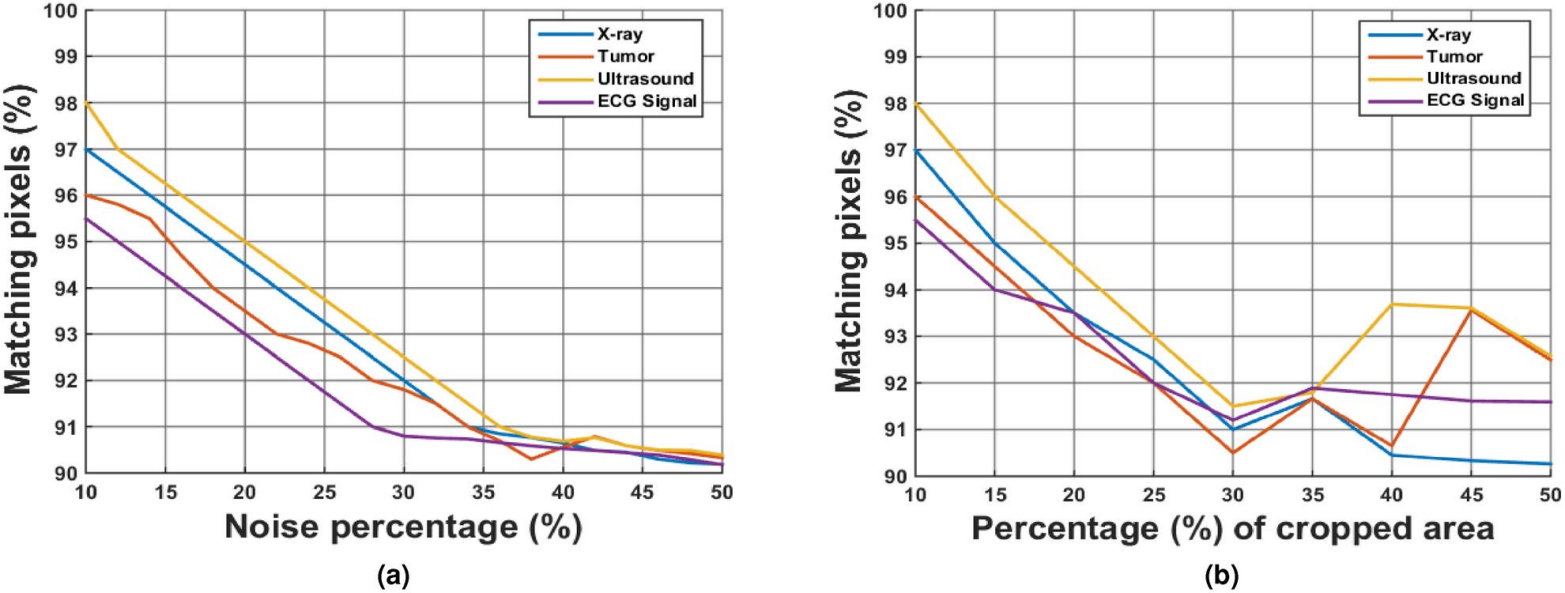
Noise and cropping attack analysis for different medical images.

Several noise and cropping curves are generated for different images to demonstrate how much plaintext data can be recovered after adding external salt and pepper noise at varying percentages from 10% to 50%, with a step size of 2%. Likewise, for the cropping attack analysis, the image is cropped from 10% to 50% in increments of 2%. Figure 9 illustrates that even with the addition of 50% salt and pepper noise to the ciphertext, the proposed framework is capable of decrypting more than 90% of the plaintext information. Similarly, after cropping 50% of the ciphertext image, over 90% of the plaintext information can still be recovered.

### Histogram analysis

Histogram analysis is a technique used to assess the security and effectiveness of an encryption algorithm by analyzing the pixel distribution in the image^48^. For an unencrypted image, the histogram shows different patterns corresponding to the visual content of the image. For an encrypted image, the histogram should be uniform and distinctly different from that of the plaintext image. This uniformity indicates that the encryption algorithm has effectively encrypted the original pixel values, which makes it difficult for an eavesdropper to extract any meaningful information.

**Fig. 10:**
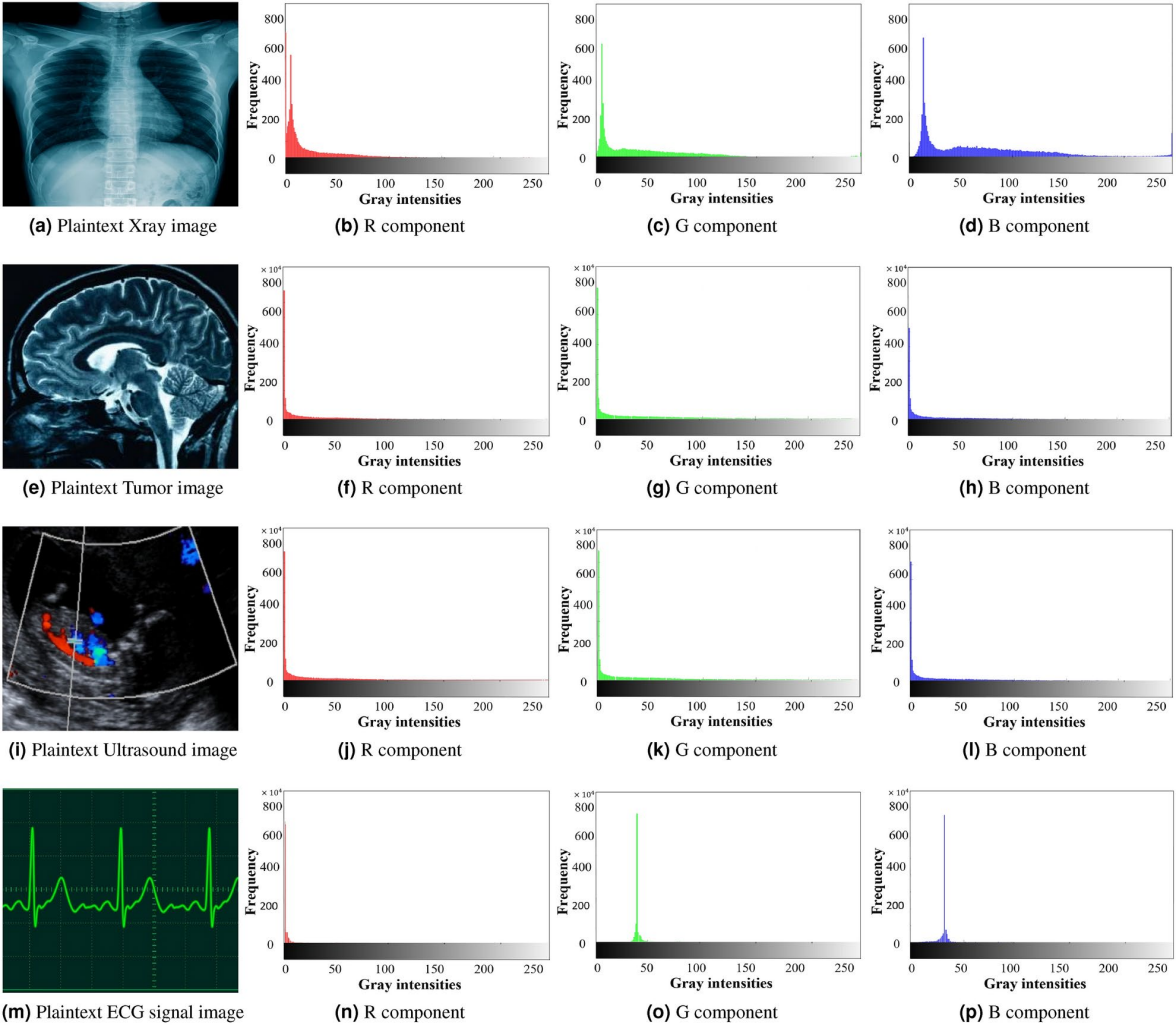
Histograms of the plaintext color images.

**Fig. 11:**
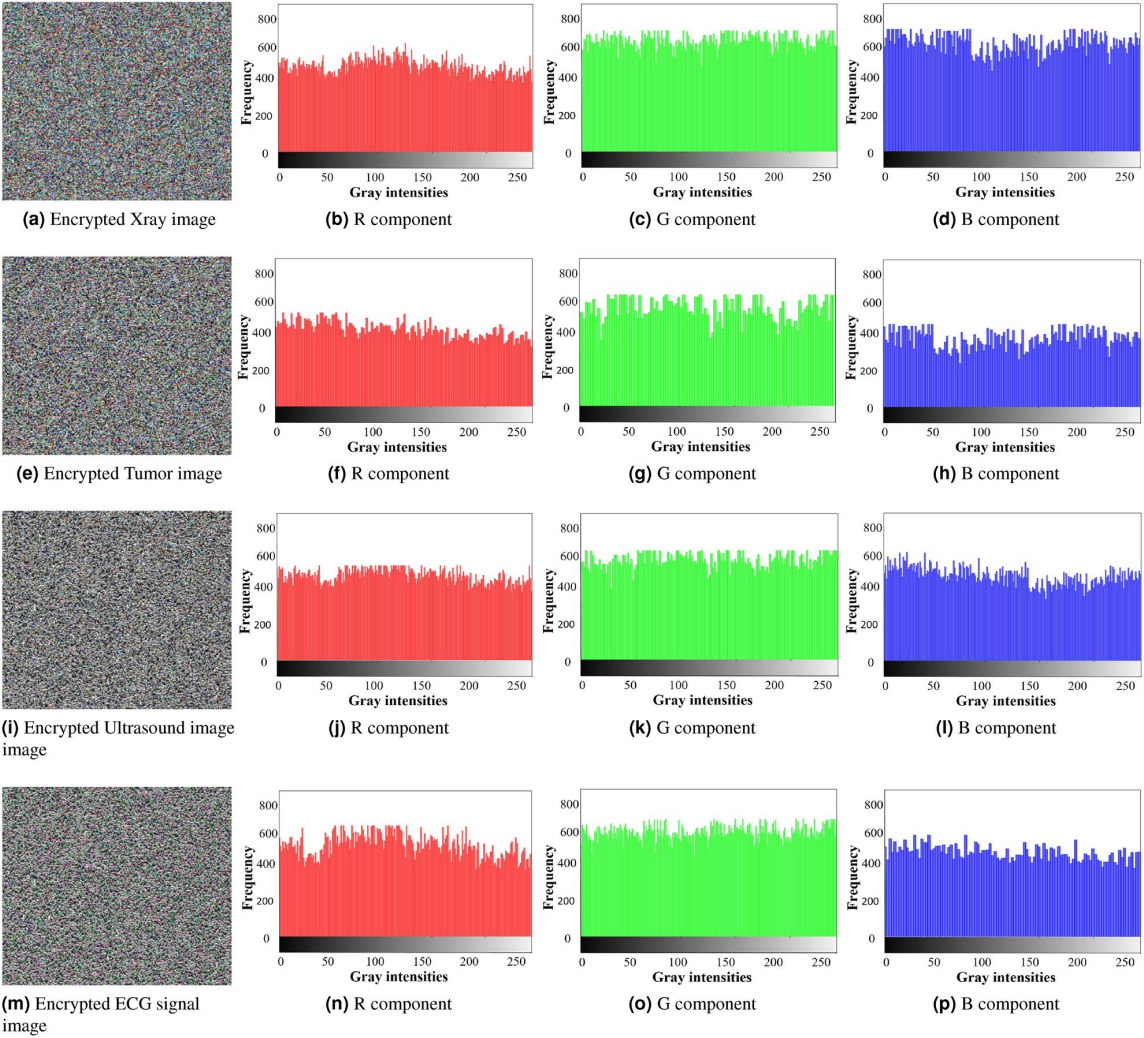
Histogram of the encrypted color components.

In the proposed research, several histograms are generated for the different color components of the images. Figure 10 displays the histograms of the unencrypted color components, while Figure 11 shows those of the encrypted color components. The histograms of the encrypted color components show a uniform pixel distribution and differ significantly from those of the plaintext color components, indicating that the proposed encryption framework effectively resists histogram attacks.

### Histogram variance analysis

Histogram variance analysis is used to statistically evaluate the effectiveness of an encryption algorithm by examining the distribution of pixel intensities in the encrypted image. Mathematically, histogram variance can be calculated using Equation 35.

35 $$\begin{aligned} \sigma ^2 = \sum _{i=0}^{L-1} p(i) \cdot (i - \mu )^2 \end{aligned}$$

Where, $\sigma ^2$ is the histogram variance, $L$ is the number of possible intensity levels, $p(i)$ is the probability of occurrence of intensity level $i$, and $\mu$ is the mean intensity of the histogram which can be calculated as $\mu = \sum _{i=0}^{L-1} i \cdot p(i)$. The histogram variance $\sigma ^2$ quantifies how pixel intensities deviate from the mean intensity value. For a strong encryption, the histogram variance should be low which indicates a uniform distribution of pixel values. Table 5 shows that the proposed encryption framework achieves lower histogram variance values than existing schemes, indicating its superior performance.

**Table 5:** variance analysis.

Image size	Images	^32^	^28^	^44^	^45^	^46^	Proposed
$256 \times 256 \times 3$	Xray	260.36	261.46	261.30	260.15	259.16	258.31
	Tumor	261.03	262.34	265.66	270.16	266.03	257.01
$512 \times 512 \times 3$	Ultrasound	266.31	261.09	270.16	267.78	260.33	259.46
	ECG signal	260.19	265.19	270.19	259.66	26011	259.88

### Lossless analysis

Lossless analysis determines whether any plaintext information is lost after decrypting the plaintext image from the ciphertext image. To assess whether the proposed encryption method is lossless or lossy, two well-known metrics are used: Peak Signal-to-Noise ratio (PSNR) and Mean Squared Error (MSE). PSNR measures the similarity between the plaintext image and the decrypted image, while MSE calculates the difference between these images. Mathematically, PSNR and MSE can be computed using Equations 36 and 37, respectively.

36 $$\begin{aligned} \text {PSNR}= & 10 \cdot \log _{10} \left( \frac{\text {MAX}^2}{\text {MSE}} \right) \end{aligned}$$

37 $$\begin{aligned} \text {MSE}= & \frac{1}{MN} \sum _{i=0}^{M-1} \sum _{j=0}^{N-1} \left( I(i, j) - K(i, j) \right) ^2 \end{aligned}$$

Where, $I(i, j)$ and $K(i, j)$ represent the pixel values at position $(i, j)$ in the plaintext and decrypted images, respectively, and $\text {MAX}$ is the maximum possible pixel value of the image (e.g., 255 for an 8-bit grayscale image). Table 6presents the PSNR and MSE values for both the proposed and existing encryption schemes. The MSE values for the encryption framework proposed in^44^ are nearly zero for each corresponding plaintext color image. In contrast, for the proposed encryption framework, all PSNR values are infinite and all MSE values are zero, indicating no difference between the decrypted image and the original plaintext image. This demonstrates that the proposed encryption algorithm is lossless.

**Table 6:** Lossless analysis.

PSNR							
Image size	Images	^32^	^28^	^44^	^45^	^46^	Proposed
$256 \times 256 \times 3$	Xray	50.5	59.12	63.32	71.65	50.3	$\infty$
	Tumor	55.61	66.15	70.64	84.64	59.16	$\infty$
$512 \times 512 \times 3$	Ultrasound	59.61	61.76	68.97	74.68	68.31	$\infty$
	ECG signal	60.19	66.97	70.64	73.15	69.11	$\infty$

MSE							
Image size	Images	^32^	^28^	^44^	^45^	^46^	Proposed
$256 \times 256 \times 3$	Xray	10.69	15.19	16.59	5.16	21.34	0
	Tumor	11.65	16.19	18.33	3.59	22.66	0
$512 \times 512 \times 3$	Ultrasound	16.11	19.49	20.97	1.69	24.09	0
	ECG signal	19.19	18.16	20.19	4.98	24.67	0

### Computational time analysis

Apart from statistical security analysis and conducting cyberattacks on the ciphertext images to assess the effectiveness of the proposed encryption framework, it is also essential to evaluate the encryption scheme’s computational complexity. For real-time applications and low-memory devices, such as IoT devices, the encryption framework must minimize the time required to encrypt plaintext images. To determine the computational complexity of the proposed encryption framework, MATLAB commands ‘tic‘ and ‘toc‘ are used to measure the elapsed time between two points in a script.

The ‘tic‘ command records the current time when it is executed, capturing the precise time in seconds since a fixed reference point in the past. In contrast, the ‘toc‘ command calculates the time elapsed since the most recent ‘tic‘ command was executed. Mathematically, if $T_{\text {start}}$ and $T_{\text {end}}$ represent the times at which ‘tic‘ and ‘toc‘ are executed, respectively, then the elapsed time $\Delta T$ is given by Equation 38.

38 $$\begin{aligned} \Delta T = T_{\text {end}} - T_{\text {start}} \end{aligned}$$

Table 7presents the computational complexity analysis for the proposed and existing encryption algorithms. It can be observed that the encryption algorithm proposed in^45^ performs slightly better than the proposed encryption framework. However, the proposed encryption framework is still suitable for real-time and IoT applications that require fast processing, as it can encrypt plaintext images of sizes $256 \times 256$ and $512 \times 512$in under one second. Moreover, apart from the encryption scheme in^45^, the proposed encryption framework outperforms other comparable encryption schemes in terms of computational complexity.

**Table 7:** Computational time analysis.

Image size	Images	^32^	^28^	^44^	^45^	^46^	Proposed
$256 \times 256 \times 3$	Xray	0.014	0.220	0.362	0.0001	0.79	0.002
	Tumor	0.016	0.259	0.038	0.0002	0.89	0.003
$512 \times 512 \times 3$	Ultrasound	0.030	0.430	0.636	0.002	1.68	0.003
	ECG signal	0.035	0.510	0.729	0.004	1.59	0.005

## Discussion

The proposed work consists of two main components: (i) securing secret keys from potential attackers using Quantum Key Distribution (QKD), and (ii) protecting plaintext medical images through the integration of multiple encryption techniques. The QKD is utilized alongside One-Time Pad (OTP) encryption to protect the secret keys, and the proposed research mathematically demonstrates that these keys are fully secured against cyberattacks. Additionally, the integration of multiple encryption techniques aims to maximize randomness in the plaintext data to enhance protection against cyber threats.

For instance, using a single encryption method, such as a permutation operation that makes the encryption vulnerable to various attacks. When only a permutation function is applied to a plaintext image, the process remains insecure, as it only rearranges pixel positions without altering their values. This means that key patterns and structural information within the original image are largely preserved which makes it easier for attackers to reconstruct the image. Moreover, a ciphertext-only attack (COA) becomes possible when only a permutation is employed. In such cases, an attacker can take advantage of the statistical characteristics of the image, including patterns in color distribution or variations in intensity, to partially or completely reconstruct the original image. One more significant weakness of a permutation-only encryption method is its vulnerability to known-plaintext attacks (KPA). When an attacker has access to both the encrypted image and its corresponding plaintext image, they can analyze the pixel rearrangements to determine the permutation pattern. This ability to exploit statistical features makes the encryption scheme highly susceptible to reconstruction efforts. Furthermore, histogram analysis also presents a significant threat to permutation-only encryption. Since permutation does not change pixel values, the histogram of the encrypted image remains identical to that of the original image. This similarity enables attackers to analyze the histogram which provides statistical clues about the structure and content of the original image. Finally, the lack of diffusion in permutation-only encryption schemes makes them particularly weak. According to Claude Shannon^49^, an encryption scheme can be considered secure only if it incorporates both confusion and diffusion operations. Therefore, the proposed encryption framework effectively integrates multiple encryption methods to create both confusion and diffusion in the plaintext data. This ensures strong security with low latency, as demonstrated by statistical security analyses, computational evaluations, and comparisons of the proposed encryption scheme’s strength with existing encryption methods presented in Section 5.

## Conclusion

This research presented a new hybrid encryption framework that combines quantum and classical cryptographic techniques for the secure transmission of medical images in IoT-based telemedicine networks. By leveraging QKD for the generation of secure shared keys and integrating classical cryptographic methods, the proposed framework addressed several security concerns such as weak key management, and weak security of the digital data. The proposed framework employed multiple encryption techniques, such as pixel shuffling, bit-plane extraction, and various transformation techniques for the enhancement of the confusion and diffusion between the plaintext image pixels. Experimental results demonstrated the robustness of the proposed encryption framework. The proposed encryption framework achieved impressive values in entropy, correlation, and key space, which are 7.999, 0.0001, and $2^{741.67}$, respectively. Moreover, the proposed framework is also tested against different cyber threats, including noise, cropping, and brute-force attack. It is found that after the addition of the external noise and cropping various portions of the ciphertext image, the proposed framework remains capable of decrypting over 90% of the plaintext image. This also showed its capability to protect sensitive medical data against unauthorized access and tampering. Additionally, the proposed encryption framework is well-suited for real-time and IoT applications where fast processing speed is required. This is demonstrated by the computational complexity analysis, where it is observed that the proposed encryption framework can encrypt plaintext color images of sizes $256 \times 256$ or $512 \times 512$ within one second.

The proposed encryption framework can be further enhanced by incorporating visual cryptography using DWT. Additionally, to further reduce computational time, Artificial Intelligence (AI) will be integrated to identify and focus on the Region of Interest (ROI) for encryption.

## Data Availability

The datasets used and/or analysed during the current study available from the corresponding author on reasonable request.
